# The Genus *Iris* Tourn. ex L.: Updates on Botany, Cultivation, Novel Niches and Impactful Applications

**DOI:** 10.3390/plants14182870

**Published:** 2025-09-15

**Authors:** Ioana Crișan

**Affiliations:** Department of Crop Science—Botany, Faculty of Agriculture, University of Agricultural Sciences and Veterinary Medicine from Cluj-Napoca, Calea Manastur 3-5, 400372 Cluj-Napoca, Romania; ioana.crisan@usamvcluj.ro

**Keywords:** medicinal, ornamental, symbolism, agronomy, phytoremediation, orris, bioactivity, crops, rhizome

## Abstract

The genus *Iris* is a perfect example of ethnobotanic continuity, where symbolic, medicinal and cultural relevance have provided foundations for many of the ornamental, phytochemical, pharmacological, industrial and ecological applications we know today. The aim of this review is to provide an up-to-date perspective on the past, present and future of this genus at the nexus of various interconnected disciplines, focusing on the most impactful and promising applications. The genus’s most economically relevant areas of importance are ornamental use and the industrial application of orris. Biodiversity studies provide a necessary basis of knowledge for preserving its valuable genetic pool; optimized cultivation technologies ensure the flow of raw materials to various industries; and scientific research opens new niches for applications. *Iris* extracts and compounds have been shown to be effective against certain human pathogens, including fungi, bacteria, and protozoa. Newly screened species indicate promising antimicrobial and antioxidant bioactivity, hinting at their unexplored potential. New compounds have been isolated and studied, exhibiting pharmacological and immunomodulatory potential. *Iris*-derived exosomes with skin-protective effects and iris extract-coated nanoparticles that can be applied in phytoremediation are among the newest findings. Novel niches explore the potential of useful microbiomes from wild-sampled irises and the use of allelochemicals as bioherbicides. Current scientific evidence supports the medicinal, ecological and industrial relevance of this genus.

## 1. Introduction

Many of today’s global challenges relate to health and well-being, environmental pollution and biodiversity loss. It is particularly fascinating that plants from a botanic genus could intersect with all of these areas. From over 300 species in the genus *Iris* [1], between 25 [2] and 30 species [3] are known to have been used in traditional medicine. Given the diversity of this genus and the large number of species never screened before, much is yet to be discovered, posing exciting possibilities for research. A large proportion of drugs are still derived from plants. Between 1981 and 2010, over 1000 new compounds were approved for pharmaceutical use, with over 50% of them being naturally derived, mostly from plants. However, it is estimated that only a small proportion of the existing phytochemical diversity of plant compounds has been explored [4]. Earth flora is a rich source of crude drugs that must be classified by various methods within pharmacopoeial definitions [5]. Comprehensive botanical and phytochemical studies can provide pharmacognostic markers essential for the unambiguous identification and quality control of *Iris* materials [6], and further bioactivity screening can provide insight into potential applications [2]. Ornamental irises are easily recognizable spring flowers, having been extensively bred and integrated into landscapes and gardens worldwide. The genetic improvement of irises remains an active pursuit of modern floriculture [7]. Industrial irises are cultivated for their rhizomes (orris), which are processed to obtain valuable essential oils for use in perfumery [8] and sold at premium rates of over 40 EUR per 1 mL [9]. However, this value may fluctuate based on quality, suppliers and other considerations. Some irises find use in environmental applications, with a reputation within the phytoremediation field as agents capable of mitigating pollutants, making them a highly sought-after green technology within the current context of increasing pollution and its threats to the health and stability of ecosystems [10]. Species of the genus *Iris* are adapted to various environments [11], some being particularly rare and understudied to date, yet studying these can give clues as to their growth potential for various purposes. Furthermore, ex situ conservation efforts are needed and in situ protection measures should be set in place to preserve their diversity [12].

There is a lack of recent work that is sufficiently comprehensive to touch on all current knowledge on this genus and reflect on its important implications. Therefore, this work invites an overview of the genus *Iris,* with emphasis on its contemporary significance. For this purpose, were gathered established and novel sources alike in order to provide a comprehensive view. This work provides a historical background on *Iris*’s symbolism and attestation of use, in addition to its botany, modern applications and future perspectives. In particular, this work highlights the most impactful findings of practical relevance by relying on the most recent published research. This thorough approach was undertaken in order to fundamentally explain why and how the genus *Iris* came to represent a group of valuable multipurpose plants.

The aim of this work was to provide an updated and integrative review of the genus *Iris* by highlighting its botanical features, cultivation practices, relevant phytochemical profile and current and emerging applications, seeking to illustrate past, present and future continuity and the interconnectedness of multiple disciplines framed into a practical perspective. Three objectives were defined to achieve this aim:

- examining the classical literature on the genus’s botany, cultivation and applications;
- assessing the phytochemical diversity and pharmacological potential, with emphasis on the most impactful applications;
- identifying trends and challenges in novel uses to outline future directions.

## 2. Cultural Importance

The *Iris* genus mentioned in the XVIII^th^-century work of Linnaeus [13] designates the group of plants known in today’s world as irises. However, Theophrastus was credited as the first to use this name in reference to these plants, and the same name also appeared in other early sources [14]. The etymology of *Iris* originates from the Greek word for “rainbow” (*Yreos*), sharing its name with an ancient Greek goddess [14,15,16,17], which was most likely chosen due to the wide variety of floral colors these plants are able to display. Various common names across cultures are used in a wide variety of languages to describe the plant. For example, the Croatian name of the plant is “perunika”, resembling the name of the ancient Slavic god Perun [18]. The iris plant was a symbol of Amesha Spenta Ameretat in ancient Iran [19]. Since ancient times, a strong association has existed across cultures between these plants and gods, death and the afterlife. As a sign of approaching death, Eurydice was represented with an iris on her collar in a mural art panel at Akrotiri [16], and these plants decorated the funeral ornaments of Syrian and Etruscan tombs [17]. Ancient Greek women planted irises on graves in the belief that they may accompany the soul into the afterlife [20]. Similar habits persisted in Turkey throughout the Ottoman Empire up to the 20th century [16,21]. *I. florentina* [*I. albicans*] and *I. sicula* [*I. mesopotamica*] were utilized in Muslim cemeteries from Spain to Kashmir [22]. Being highly valued for its curative and magical properties since ancient times in Europe, the harvesting of iris rhizomes involved specific ritual [20]. *Iris* was used in a ceremonial bath in parts of South China or hung on doors for protection [23]. In ancient Japan, the blue iris signified bravery [16].

In French historical lore, the iris flower has been a symbol of Gaul since as far back as the 1st century AD [24]. *I. pseudacorus*, growing near waters with a display of yellow flowers, is associated with the legend of Clovis the First, a fifth-century king of the Franks and founder of the Merovingian dynasty [24,25]. The golden iris (*fleur*-*de*-*lis)* on a blue background remained the heraldic symbol of the French monarchy until its end and is its most recognizable symbol to this day. Throughout history, the *Iris* flower was a consecrated symbol of chivalry and became a heraldic emblem across many countries between the fourteenth and nineteenth centuries, from Hungary to Italy and Britain [16].

In Late Medieval Europe, the iris flower was adopted as a symbol of purity by Christianity and associated with the Virgin Mary, as observed in famous sacred artworks such as “The Virgin and Child in a garden” (c. 1469–1491) by Martin Schongauer and “Madonna with the iris” (c. 1500) by Albrecht Dürer (The National Gallery, London, UK). In the Middle East, iris plants were depicted by artisans in famous Zarrinfam pottery from the Safavid era (XVI to XVIIIth centuries), often accompanying the poppy in a complex symbolism [19]. These flowers continued to inspire later famous artistic masters such as Van Gogh (Van Gogh Museum, Amsterdam, Netherlands) and Claude Monet (The National Gallery, London, UK) in their depictions.

Several *Iris* species are still celebrated as spring heralds at Nowruz Festivals in Anatolia, Central Asia, and the Caucasus [26]. The city of Florence in Italy retains the *Iris* flower as its emblem [17]. Today, this symbol is still used as a logo by various brands and organizations to evoke legacy and heritage.

## 3. Botany

### 3.1. General Overview

At the time of writing, 313 recognized species are listed under the genus *Iris* [1], belonging to the family Iridaceae. The age of this botanic family inferred based on its molecular clock (due to a current lack of fossils of significant age) places its origin in the Cretaceous, with an age of about 80 million years [27]. Today, the genus is recognized as a temperate group of deciduous or evergreen perennial plants that diversified in the mesic and xeric habitats along the temperate Northern Hemisphere, with high-diversity centers around the Mediterranean Basin and in Asia [11,28]. Distinctive from other genera of the family Iridaceae, *Iris* presents a petaloid style and perianth whorls of varying morphology. Within the genus, variations in geophytic organ, lower tepal ornamentation and seed characteristics can be observed across six subgenera [11,28,29].

**Figure 1:**
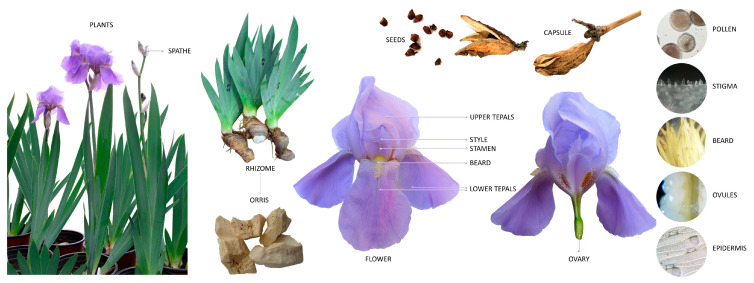
Morphological features of a cultivated bearded species: *I. pallida* (original by I.C.).

Its morphological and histo-anatomical aspects are key pharmacognostic features, essential for the correct identification and quality control of *Iris* species with medicinal relevance [6]. The general morphological features of *I. pallida*, one of the commonly cultivated species for industrial purposes, are presented in Figure 1. Notable characteristics of this species are visible: thick rhizomes, ensiform leaves, flower buds protected by spathes with papery consistence, pale-purple flowers, lower tepals with hairs (beard) and fruit represented by a pod (capsule) with brown seeds inside.

### 3.2. Organography: Current Overview and New Insights

#### 3.2.1. Vegetative Organs: Root, Storage Organs and Leaf

**Figure 2:**
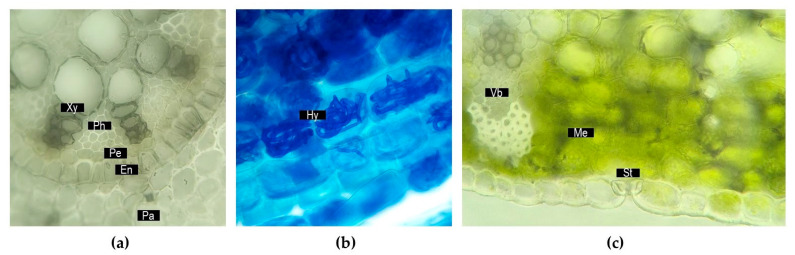
Histologic features of vegetative organs in *Iris*: (**a**) Details of actinostele in root of *I. germanica.* Pa—parenchyma tissue of the root cortex; En—endodermis with a “horseshoe” aspect of cells due to thickened cell walls; Pe—pericycle where lateral rootlets originate; Ph—phloem; Xy—xylem. (**b**) Arbuscular mycorrhiza colonization of *I. sibirica* cortical cells displaying hyphae coils. Hy—hyphae. (**c**) Leaf section of *I. pallida.* Vb—vascular bundle; Me—mesophyll chlorenchyma; St—stomata (original by I.C.).

The vegetative organs of *Iris* plants present notable characteristics and adaptations depending on species [11]. *Iris* has been used as a model for the primary root structure of monocots in botany books, with *I. germanica* being the primary example in this regard [30,31]. The presence of a multiseriate exodermis in *I. germanica* roots has been suggested as a potential adaptation of the plant to periods of drought, having been employed as a model in root permeability studies [31]. The *Iris* root presents three main histologic zones—the rhizodermis, cortex and central cylinder—with vascular tissue arranged in an actinostele, as shown in Figure 2a [32].

Studies in recent decades have demonstrated the ability of *Iris* roots to establish symbiotic relationships with arbuscular mycorrhizal fungi, as described for *I. germanica* [33], *I. sibirica* [34] and *I. pseudacorus* [35] to name a few. These fungi enhance phosphorus uptake by the plant and proliferate in *Iris* cortical cells following a *Paris*-type pattern [36], in which hyphae coils pass from cell to cell (Figure 2b).

The root has been proven to be an important site for the remediation of pollutants. A recent study demonstrated that inoculation of *I. tectorum* roots with *Rhizophagus irregularis*, a commonly studied arbuscular mycorrhizal fungus, resulted in enhanced As (arsenic) translocation in addition to the mitigation of phytotoxicity symptoms in the plants under hydroponic conditions [37]. Studies also proved the beneficial effects of arbuscular mycorrhizal fungi on *I. tectorum* under Cr (chromium) contamination [38,39]. Focus is placed on the roots due to the practical implications of the interactions and mechanisms in the rhizosphere regarding efficiency in mitigating increasing levels of environmental pollutants.

**Figure 3:**
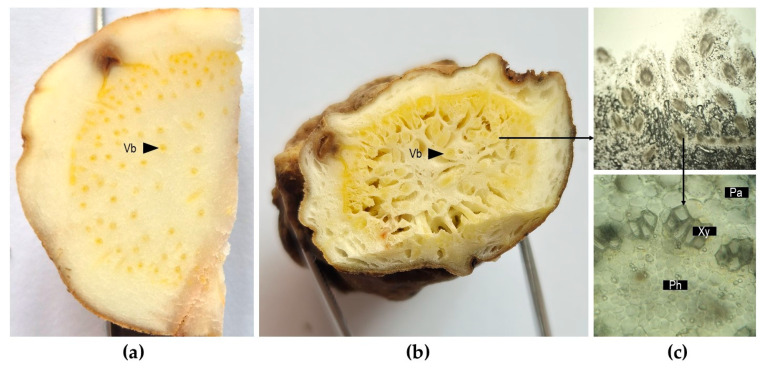
Transversal section of *I. pallida* through a young rhizome (**a**) and old rhizome (**b**); microscopic view of leptocentric vascular bundles inside the rhizome (**c**). Vb—vascular bundles macroscopically visible as yellow dots; Pa—parenchyma tissue. Vascular tissue: Xy—xylem; Ph—phloem (original by I.C.).

Irises present modified underground organs that store nutrients to ensure the perennity of the plants. Geophytic organs include rhizomes, tuberous roots and bulbs [28]. A study on bulbous *Iris* species (*I. bucharica*, *I. reticulata*, *I. xiphium*) showed that bulbs mainly store starch and fructans in addition to glucose, fructose and sucrose at low levels [40]. The morphology of the rhizome can vary with species in this genus, ranging from horizontal to oblique and erect, from thick to thin and more or less branched [11]. The most important species for orris production (*I. florentina* and *I. pallida*) present thick fleshy rhizomes that grow horizontally at shallow depths. The rhizome in these species is collected for medicinal and fragrant use [41]. A histologic examination of the rhizome showed well-developed parenchymal tissue in the interior that stores organic compounds (e.g., starch in *I. germanica* or fructans in *I. pseudacorus*) [14]. A large number of leptocentric vascular bundles are scattered within the parenchyma [14,42], as shown in Figure 3a–c.

*Iris* leaves are usually unifacial, ensiform or linear [11]. Few species are evergreen, and most are deciduous. Anatomic observation of an ensiform-type leaf reveals mesophyll with chlorenchyma concentrated under both sides of the epidermis, lacunose tissue located at the center of the leaf and collateral vascular bundles under each side enforced by thickened cells (sclerenchyma) towards the exterior for mechanical strength (Figure 2c). Environmental factors intervene in the natural onset and progression of leaf senescence in *Iris,* which is related to dormancy during unfavorable seasons (usually winter, in a few species during summer). Cold stress affects the survival, growth and flowering in the next vegetative season. One study indicated that interspecies differences in the leaf freezing tolerance of irises depend on the rate and degree of cold acclimation, which activates signaling networks that involve the transcription induction of cold-responsive genes. Histological differences have been described in evergreen (*I. hexagona*) and deciduous (*I. pseudacorus*) irises that could be explained by their habit [43]. In addition to cold tolerance, which has been considered in establishing the hardiness scale [44], a novel challenge is adaptations to temperature fluctuations associated with global warming, which threatens the ecologic stability of many species today. Increases in temperature under experimental conditions caused physiological and structural adjustments in *I. pumila* related to thermal stress-coping mechanisms. Thermal stress in *I. pumila* also induced a decrease in specific leaf area and stomatal density, while chlorophyll content displayed the highest plasticity out of the studied traits, suggesting that growth and metabolic activity have priority over structural investment under increasing temperatures [45]. Less is known regarding how changing climatic trends impact geophytes such as *Iris* in regard to their dormancy.

#### 3.2.2. Generative Organs: Flower, Fruit, Seed

The majority of *Iris* species flower from spring to summer, with the exception of species from the series *Unguiculares* (subgenus *Limniris,* section *Limniris*) that flower from autumn to spring [29], in addition to certain bearded hybrid rebloomers. Spring flower displays are the reason for these plants’ widespread ornamental use. The flowers have three reflexed tepals, called falls, and three upward-oriented tepals, called standards. Three stamens and three petal-like styles arch over lower tepals [11,29]. Flowers either emerge solitary or in inflorescence (rhipidium). Each flower acts as a meranthium, with the appearance of a compound flower consisting of three pollination units that, to pollinators, resemble individual flowers. Each of these units provides pollinators with a platform consisting of the limb of the outer tepal (syn. sepal). Most species secrete small quantities of nectar as a reward for pollinators [27]. Nectary tissue is located inside the perianth tube, preventing certain pollinators from reaching it [46]. Hymenopteran bees are the primary pollinators and obtain nectar, pollen and shelter from the flowers, amongst other rewards. Since the genus *Iris* is widespread, with a wide diversity of habitats in which to find them, the relationship with pollinators is sometimes particular in nature [47]. Such is the case of the Royal irises (subgenus *Iris* section *Oncocyclus*) spread in the Middle East, which lack nectar and provide overnight shelter to the male *Eucera* spp. that pollinate them [48], while some Louisiana irises (section *Iris*, series *Hexagonae*) such as *I. fulva* are pollinated by hummingbirds [49].

A recent study on key genes and the pathways involved in regulating flowering in *I. dichotoma* and *I. domestica* showed that genes promoting cellular water uptake, cell wall loosening and osmotic pressure regulation were up-regulated upon flower opening, causing an increase in turgor pressure in cells [50]. A study on two *I. germanica* cultivars identified *IgUBC* and *IgGAPDH* as stable reference genes involved in flower bud development [51]. Reblooming in bearded irises is a highly sought-after trait that involves the expression of several flowering-related genes. Further insights provided evidence that the *IgFT* gene may play an essential role in the second floral initiation of bearded irises [52], hinting at possibilities for molecular breeding of reblooming irises. Moreover, the flowering repressors *IgSVP* and *IgTFL1* may prove instrumental, and knocking out such repressors via CRISPR/Cas9 could make it possible to extend the decorative season of bearded irises [53].

**Figure 4:**
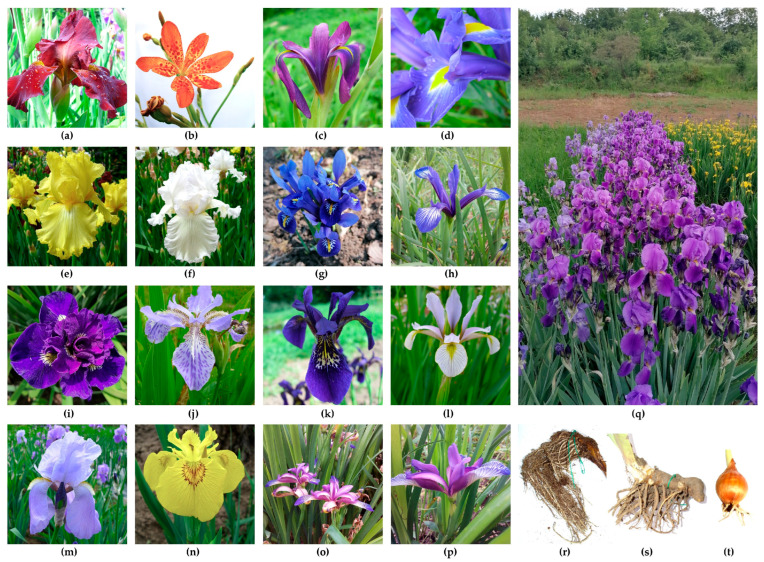
Examples of morphologic diversity in *Iris*: *I. germanica* ‘Sultan’s Palace’ (**a**), *I. domestica* (**b**), *I. ensata* var. *spontanea* (**c**), *I. × hollandica* (**d**), *I. germanica* ‘Lime Fizz’ (**e**), *I. germanica* ‘Pure As The’ (**f**), *I. reticulata* (**g**), *I. sintenisii* (**h**), *I. sibirica* ‘Concord Crush’ (**i**), *I. tectorum* (**j**), *I. chrysographes* (**k**), *I. halophila* var. *sogdiana* (**l**), *I. pallida* (**m**), *I. pseudacorus* (**n**), *I. pseudocyperus* (**o**), *I. colchica* (**p**), *Iris* sp. crop (**q**), *I. sibirica* rhizome (**r**), *I. germanica* rhizome (**s**) and *I. × hollandica* bulb (**t**) (original photos by I.C.).

Once open, irises provide colorful displays with ornamental value, but they also play a role in mediating interactions with pollinators. The presence of colors in irises (Figure 4) occurs due to the presence of anthocyanin pigments (creating pink, purple, or red colors) or carotenoids (as seen in orange and yellow flowers) or an absence of pigments (in white or creamy flowers) [54]. The characterization of pigments extracted from purple *Iris* sp. flowers indicated the presence of the five anthocyanin aglycones: cyanidin, delphinidin, petunidin, pelargonidin and malvidin [55].

A study on *I. sanguinea*, which aimed at revealing key genes and metabolite networks that cause pigmentation in flowers, revealed the occurrence of a gradual increase in the expression of a large set of genes involved in flavonoid and anthocyanin biosynthesis pathways. These further correspond to a gradient of anthocyanin accumulation and, ultimately, different color phenotypes [56]. While differences between some colors are striking, differences between purple, violet and blue are often vague among irises. Blue polymorphisms among species such as *I. laevigata*, *I sibirica* [*I. typhifolia* and *I. sanguinea*] and *I. lactea* arise due to different proportions of delphinidin pigments accumulated in flowers [57]. A more in-depth study on *I. potaninii* from the Qinghai–Tibet Plateau, which presents both blue and yellow flowers, suggested that yellow flowers, which are more attractive to pollinators, occur due to a loss of anthocyanins, and *F3H* could be a key gene involved in this process [58]. A better understanding of the processes involved in color phenotypes among irises in different environments may enable the modeling and prediction of their adaptive responses.

Some species such as *I. fulva* lack scent, but most irises present scented flowers [27]. A comprehensive screening of a large number of accessions belonging to the most cultivated bearded species (*I. germanica*, *I. pallida* and *I. pumila*) identified over 200 scent components, with 34–64 per accession, the major components being caryophyllene, linalool, citronellol, methyl cinnamate, β-cedrene, thujopsene, methyl myristate, linalyl acetate, isosafrole, nerol and geraniol [59]. Another study evaluated the variation in volatile components (alcohols, aromatics and aldehydes) released by flowers of *I. uniflora* and *I. sibirica* [*I. typhifolia* and *I. sanguinea*]. It was determined that nonyl aldehyde contributes substantially to the scent of these species, while a variation in emitted compounds exists across flowering stages [60]. A more in-depth molecular investigation into *I. germanica* indicated that the gene expression level of *IgTPS14* was related to linalool release from flowers [61].

The lower tepals can present a patch of hairs, such as in *I. germanica* (Figure 4a,e,f) or *I. pallida* (Figure 4m), which is referred to as a beard. This aspect is missing in beardless species, yet distinctive markings may still be visible, such as patches of colors or veins as seen in *I. sintenisii*, *I. chrysographes* and *I. pseudacorus* (Figure 4h,k,n). A crest might be present on the lower tepals, such as in *I. tectorum* (Figure 4j).

Iris pollen is sulcate, with some palynological features that might be useful in terms of taxonomy. While pollen morphology has been used in the phylogeny of Iridaceae, infrasubgeneric studies in *Iris* remain scarce [62]. The flowers have an ovary, which is usually three-locular (or rarely one-locular (*I. tuberosa*)) [27] and, after fertilization, produces fruit in the form of a 3–6-angled pod (botanically classed as a capsule) [29]. Despite not having been studied in detail, the natural variations in seed morphology may be associated with certain adaptations regarding seed dispersal. The bright-colored seeds of *I. foetidissima* may be dispersed by birds, while *I. ruthenica* seeds, which have a white elaiosome, have been documented to be dispersed by ants. Furthermore, *I. unguicularis* seeds are covered with glands that attract ants. *I. pseudacorus* seeds float for many months and can be dispersed by water until they finally sink and germinate [27]. The seed can present a terminal aril or may lack one entirely [28], a fact that has taxonomic significance.

Botanic characterization is important for identifying and classifying species, as well as for providing the required diagnostic pharmacognostic features for their safe use. Hence, this is a fundamental basis of knowledge for scientific research on this genus from both theoretical and practical perspectives. In addition, certain physiological aspects remain relevant in terms of the ecology of species, as well as for their cultivation and conservation.

## 4. Classification and Insights into Species

### 4.1. Classification of Irises

Two main approaches are used to classify irises. The first is the current scientific classification, grouping them according to their morphology and phylogenetic relationships into subgenera, sections and series, which is important for botanists and breeders alike. The second is horticultural classification, which places irises into groups according to ornamental phenotypes and their purpose in garden settings, a more important approach for gardeners and the general public. However, the two approaches are not entirely separate, as the horticultural classification relies somewhat on the botanic classification.

*Iris* genus taxonomy is not without its challenges due to discrepancies between molecular and morphologic data in relation to phylogenetic inferences [63]. According to the Iris Working Group in the updated work of White et al. [11], a generally accepted botanical classification of the genus *Iris* is presented, which recognizes six subgenera (*Iris*, *Limniris*, *Nepalensis*, *Xiphium*, *Scorpiris* and *Hermodactyloides*) with varying numbers of sections and series. In the following summary, the profiles of these subgenera are briefly described based on the most recognized sources in the field, to which the corresponding horticultural information was added, as presented in the latest Encyclopedia of Garden Plants by the Royal Horticultural Society (RHS) and other relevant sources for a general harmonized outlook on this genus that blends the two approaches.

Subgenus *Iris* comprises species with stout surface rhizomes, beards on the lower tepals, and seeds with arils (sections *Psammiris*, *Oncocyclus*, *Regelia*, *Hexapogon*, *Pseudoregelia*) [11], such as the well-known species *I. humilis* [64], as well as those without arils, as in section *Iris* where most of the cultivated garden species today are placed, such as *I. germanica*, *I. pumila* and *I. pallida*. The species of this subgenus are widespread, growing from the Atlantic coast of Southern Europe to Central Asia [11]. In section *Iris*, the horticultural classification distinguishes several groups created through hybridization and selection, such as miniature dwarf bearded (MDB), standard dwarf bearded (SDB), intermediate bearded (IB), miniature tall bearded (MTB), border bearded (BD) and tall bearded (TB) irises [29]. The creation of these bearded cultivars is presented in previous works [65]. The section *Oncocyclus* is also referred to as the Royal iris, being spread in the Middle East, with a distinctive dark spot on the lower tepals (called the signal) and characteristic visible veins on the entire flower. Some species feature particularly dark colors, such as *I. nigricans* [66]. This category has a high endemism rate [67,68], with several species currently endangered/critically endangered [69]. In addition, in this subgenus, the horticultural classification mentions Regeliocyclus as hybrids created between sections *Regelia* and *Oncocyclus*. Furthermore, hybridizing species from either of the two prior sections with another bearded species gives rise to the horticultural group Arilbred [29].

The subgenus *Limniris* comprises species with underground rhizomes and lower tepals that are smooth and beardless or sometimes crested or with fine papillae, while the seeds lack arils [27]. It includes sections *Lophiris* and *Limniris* with many series (*Chinenses*, *Vernae*, *Ruthenicae*, *Tripetalae*, *Sibericae*, *Californicae*, *Longipetalae, Laevigatae*, *Hexagonae*, *Prismaticae*, *Spuriae*, *Foetidissimae*, *Tenuifoliae*, *Ensatae*, *Syriacae*, *Unguiculares*). These species are found in Europe, Asia and North America [11]. Some particularities are notable, as in the case of *Unguiculares,* which develop a clump of rhizomes above-ground compared to species from the rest of the series with an underground rhizome. For horticulturists, the *Californicae* are known as Pacific Coast irises; *Lophiris* are known as Evansia or crested irises due to the presence of a crest or ridge on the lower tepals; *Spuriae* are known as Spuria irises; *Laevigatae* are referred to as water irises, including the yellow flag (*I. pseudacorus*), a very common species in ponds and lakes, and Japanese irises, which refer to incredibly diverse *I. ensata* cultivars with large flowers [29]. The series *Sibericae* features two subseries: *Sibericae* and *Chrysographes* [70]. The first is also known as Siberians, with *I. sibirica* as the most widespread Eurasian species, and its cultivars are widely used as garden ornamentals, displaying floral morphologic variability, yet not as widely as cultivated bearded cultivars. The latest revision synonymized *I. sanguinea* and *I. typhifolia* with *I. sibirica* [71].

Subgenus *Nepalensis* presents plants with vestigial rhizomes, tuberous roots and seeds with arils [27]. These are commonly referred to as Himalayan irises due to their distribution, which is limited to northwest Yunnan. A few species are included here, such as *I. barbatula* [11]. These have minor relevance for cultivation.

Subgenus *Xiphium* presents bulbs covered by cataphylls and seeds without arils [27]. A recent study on the relationship patterns among species of this group of Mediterranean taxa based on nuclear markers suggested the importance of the Strait of Gibraltar as a natural genetic exchange barrier [72]. Spanish, English and Dutch irises are included here [29] and, amongst these, *Iris* × *hollandica* (the Dutch iris) is the most commercially important as a cut flower crop. Its horticultural name suggests its cultivation history, which can be traced back to the Netherlands, having been created based on a contribution from four of the seven bulbous species from this subgenus: *I. xiphium, I. tingitana*, *I. filifolia* and *I. latifolia* [73]. The species of this subgenus are found in the Iberian Peninsula and Northern Africa [11].

Subgenus *Scorpiris* comprises plants that have either bulbs or swollen tuberous roots; seeds in some species have arils [27]. Horticulturists refer to them as Juno irises [29]. These species are native to Western and Central Asia. Few are cultivated for ornamental purposes, such as *I. magnifica* [11].

Subgenus *Hermodactyloides* presents bulbs with netted tunics; its leaves are cylindrical and its seeds have arils [27]. The species are found in Western Asia and Central Asia [11], referred to as Reticulata irises by horticulturists [29], the most cultivated being *I. reticulata,* with several cultivars sold in many stores, especially in temperate/colder climates, as garden ornamentals.

### 4.2. Status of Iris Species

Diversity studies and conservation measures (in situ and ex situ alike) are needed for this genus. The current trend of *Iris* plant populations varies widely. Some species are listed as critically endangered (seven species) or endangered (ten species) by the International Union for Conservation of Nature [69]. By contrast, other species such as *I. pseudacorus* have become invasive in certain areas [74], indicating the need for divergent management strategies according to species. Recent studies in diversity hotspots continue to provide important updates on the genus *Iris*, highlighting the relevance of modern botany works in completing our documentation on this genus. As such, between 2012 and 2022, seven new species were identified in Central Asia alone [75]. More research in other biodiversity hotspots is expected to provide new insights.

With increasing anthropogenic activities, intensification of the geographic fragmentation of modern landscapes and extending agricultural land, gradual habitat reductions are placing unprecedented pressure on *Iris* species populations that once inhabited undisturbed larger areas. Recent studies on endemic *Iris* species seek to untangle the genetic [76] and environmental [77] factors that influence their performance under current constraints. Such studies are essential in devising robust monitoring and conservation strategies. Furthermore, studies that can leverage indigenous knowledge [78] and foresee the future of species are particularly useful. This includes opportunities in relation to the reintroduction of taxa, as proposed for the Nazareth iris (*I. bismarckiana*) [79], or translocation, as proposed for *I. atrofusca* [80], a species with a poorly known historical distribution. The challenges in the conservation of *Iris* are as complex as this genus is diverse.

Today, botanical gardens and seed banks act as extremely important living repositories of the collections they curate and provide the most significant support for the ex situ conservation of plant species [81]. A search in the Botanic Garden Conservation International (BGCI) public data base indicates 9075 *Iris* spp. genotypes (excluding synonyms but including cultivars) registered as part of collections in botanical gardens around the world, with large numbers of species and cultivars alike. Out of these, 80 taxa are threatened or possibly threatened taxa according to the BGCI [82]. These botanical gardens are well equipped to extend *Iris* collections and contribute to their effective conservation. However, a good understanding of their needs is essential for establishing successful living plant collections, as are new and optimized cultivation technologies. This can be particularly challenging, as rare species most in need of conservation can have particular requirements, as will be discussed further.

## 5. Cultivation

### 5.1. Cultivation History

It cannot be ascertained with clarity when these plants became cultivated. As ancient sources mention their use, we can be sure of their familiarity, but it is not always entirely clear how these were sourced. One can speculate, as was the case for many useful plants, that, for practical reasons, people found it advantageous to have them in proximity [83,84] and, therefore, they might have started cultivating them for convenience [85].

Early evidence suggests that during the time of Thutmosis III, iris plants were brought to Egypt from the Near East to be grown in pharaoh gardens, a fact supported by the flowers engraved on temple walls at Karnak [20], which resemble species from section *Oncocyclus* of the subgenus *Iris*, which are commonly called Royal irises and grow throughout the Middle East [86]. From ancient times, Arabs cultivated *I. albicans* (syn. *I. florentina*). *I. trojana* and *I. cypriana* (both now recognized in *I. germanica*) as well as *I. junonia*, which bear the names of the important Greek colonies in which they were discovered. However, their establishment in habitats very different from their place of origin, in addition to their low population density, has led to the conclusion that their presence there was likely due to introduction in earlier times [14]. Several works of medieval origin mention irises [14,23], while the species *I. germanica* and *I. florentina* were grown in the countryside and monastery gardens [17]. Italy preserved an important and recognized tradition for the cultivation of *I. pallida* [87]. Notably, this species is the basis of industrial orris in Tuscany, and other species (such as *I. germanica*) are also cultivated for these purposes, mostly in other countries. Bini and Maugini, cited by the Iris Working Group, presumably identified the majority of the Tuscan *I. pallida* to be male-sterile plants, displaying a distinct karyotype and morphologically presenting a shorter stem and paler flower color, which bloom earlier than the fully fertile genotypes [11]. If this older finding is validated today, this might imply that the traditionally presumed higher orris quality obtained in Italy might also be due to a distinctive genotype.

In recent centuries, irises have been cultivated almost everywhere. The United States of America became a place for the extraordinary development of many ornamental cultivars known today thanks to the dedicated contribution of famous hybridizers such as Schreiner and many more who followed [65].

The successful cultivation of these plants depends on ornamental and medicinal applications, among others. Hence, the optimization of methods across the technological chain and the adequate management of intervening factors that impact quality represent current research foci.

### 5.2. Genetics and Breeding Objectives

In the genus *Iris*, both diploidy and polyploidy are common, with *x* = 12 and 10 being the most common base numbers [27]. Genome size dynamics in *Iris* are likely related to evolutionary processes such as speciation events [88]. Multigene factors and environmental interactions also play important roles in the biosynthesis of secondary metabolites of interest [89]. Genomes and transcriptomes are important for understanding the biosynthesis of valuable compounds by *Iris* plants and provide a basis for improvement [90]. Improvements in the germplasm of medicinal *Iris* plants target the enhancement of biosynthesis and the content of valuable compounds [91]. Today, thanks to advanced molecular techniques, sequencing data are available for seven *Iris* species, including *I. pallida* [92].

*Iris* breeding programs aim to create highly ornamental cultivars with enhanced traits. The International Union for the Protection of New Varieties of Plants (UPOV) provides testing guidelines and characteristics applicable only to bulbous *Iris* genotypes [93]. Descriptions and definitions for all horticultural groups of irises with their detailed characteristics can be found in the guidelines used for assessing new cultivars at shows, such as the handbook provided by the American Iris Society [94].

In horticultural groups of irises, tetraploids are important contributors. A recent study on the interspecific *I.* × *norrisii* reports a new polyploid germplasm obtained through the treatment of pre-germinated seeds with 0.05% colchicine, proving that, in just a short timespan, this simple method can generate genetically diverse progenies for ornamental use [95]. A further study on this genus proposed that incompatibility in interspecific hybridization occurring during the development of the embryo may be caused by low levels of indole-3-acetic acid and cytokinin. Therefore, identifying the precise phytohormone balance might allow us to obtain in vitro media for the growth of embryos resulting from interspecifically incompatible crossings [96].

The possibilities provided by biotechnology approaches in *Iris* breeding have been explored in-depth in recent work, with its many advantages laid out, such as pest and disease resistance, herbicide resistance, and new ornamental traits [7]. However, the migration of genes from genetically modified plants into non-modified species remains a general concern for transgenic plants, which could result in unintended ecological consequences; these plants have wild populations that might accidentally cross-pollinate with them, a fact that is of lesser concern in crops without close wild counterparts in the same region. Furthermore, while enhancing the resilience and toxic element uptake potential of these plants might seem an attractive route for phytoremediation purposes, one has to assess the risk of giving rise to superweeds. Considering that *I. pseudacorus* used for phytoremediation is invasive in certain regions [74], balancing all long-term ecological considerations might be the best approach.

### 5.3. Environmental Factor Requirements

The requirements for the main environmental factors—water, light, soil and climate—are best documented for the main categories of importance: bearded species for garden settings and water irises and bulbous species as cut flowers. However, having an overall understanding of the conditions experienced by various *Iris* species in their natural habitats can also be useful in devising or optimizing their cultivation technology (Table 1). Hardiness is particularly important to clarify in order to determine whether a species can overwinter successfully in more northern climates [97].

The medicinal bearded *Iris* species (*I. florentina*, *I. germanica*, *I. pallida*) are hardy (H6-H7) (Table 1) and can overwinter well in most regions of Europe with temperate climates, yet the best results are obtained in milder climates. Regarding light, these species are more exigent, since they do not develop as well in shade. They can grow in various types of soil as long as they are well drained; otherwise, the rhizome rots [98].

### 5.4. Crop Establishment

#### 5.4.1. Propagation by Seed

Seed dormancy has long been familiar to *Iris* growers and breeders [99]. *Iris* plants feature dormant embryos and germination inhibitors located in seed endosperm, which prevents germination [100]. Studies on *I. aphylla* indicated that a high abscisic acid/gibberellic acid ratio (ABA/GA) in the endosperm and accumulation of salicylic acid are responsible for poor germination [101]. A study on seed dormancy-breaking methods in *I. laevigata* indicated that after cold stratification at 5 °C for 12 weeks and incubation at 25 °C for 4 weeks, a germination rate of 51.7% was obtained, compared to a lack of germinating seeds in the absence of treatment [102]. A study on *I. mandshurica* identified that seed sterilization with AgNO_3_ and sowing of mature seeds on Murashige and Skoog medium with zero sucrose (MS^0^) with pretreatment of H_2_SO_4_ led to 80% germination within 13 days. This can be explained by the sequence of sterilization and chemical scarification provided by these substances [103]. In *I. pseudacorus*, alternating temperatures caused a high germinability rate of about 90%, compared to other experimental treatments that induced only up to 25% germination [104]. Therefore, while irises are known to be rather difficult to propagate from seeds, recent experiments have evidenced possible increases in the germination rate as a result of certain treatments, with potential for application in mass propagation. Gaining a better understanding of the modulating effect that environmental conditions have on germination [105] and optimizing seed propagation [106] remain important in the conservation efforts of certain *Iris* taxa.

**Table 1:** Relationship with environmental factors for the main *Iris* groups of current interest for cultivation.

Main Trait		Horticultural Identifier ^1^	Botanic Identifier (Genus *Iris*) [11]	Natural Habitat Conditions	Requirements in Culture and Examples ^2^
Rhizome	Bearded	Bearded species and cultivars 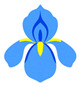 (highly cultivated for gardens, the most bred group)	subg. *Iris*, sec. *Iris*	Usually found in open habitats and rarely in high mountains, they grow in dry conditions, with some rarely in seasonal damp soil. Distribution: Atlantic Coast of Southern Europe to Central Asia [11].	Well-drained, fertile, neutral to slightly acid soil in full sun. In greenhouses in deep pots with loam-based substrate with grit, full light and low humidity, moderate watering and dry during dormancy [29]. Species: *I. germanica* (H6), *I. aphylla* (H7), *I. florentina* (H6), *I. pallida* (H7).
		Royal irises 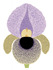 (high interest for collections/hybridizing)	subg. *Iris*, sec. *Oncocyclus*	Hot summers, rain/snow in winter and wet warm spring. There is great variation in habitats they grow in: from desert to cold steppe and high mountains. Distribution: East Mediterranean, Middle East [11].	Difficult to cultivate: they require dormancy in summer (must be kept hot and dry). Preferably in greenhouses, raised beds, or protected frames [11]. Species: *I. susiana*, *I. atropurpurea*, *I. iberica* subsp. *elegantissima* (H4).
		Regelia and Regeliocyclus 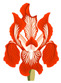	subg. *Iris*, sec. *Regelia* and hybrids with sec. *Oncocyclus*	High open mountainsides. Cold winter, wet spring and autumn, dry summer. Distribution: Central Asia [11].	Too vigorous for pot culture, suitable for plant frame/alpine house and outdoors with good drainage. Require warm and dry dormancy. Rocky substrate, granite grit, limestone [11]. Species: *I. stolonifera*, *I. afganica* (H4).
		Arilbred	hybrids (complex)	-	Loam-based potting compost with grit [29].
	Beardless	Pacific Coast natives 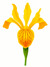	subg. *Limniris*, sec. *Limniris*, ser. *Californicae*	Adapted to mountain and forest areas, often growing in light shade. Distribution: Pacific Coast of North America [11].	Lime should be avoided in the substrate. Mulching over winter might be needed [11]. Species: *I. innominata*, *I. douglasiana* (H6).
		Siberian 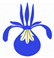 (highly cultivated)	subg. *Limniris*, sec. *Limniris*, ser. *Sibericae*, subser. *Sibericae*	Moist pastures, seepage zones. Distribution: Europe and Asia [11].	Thrive in open garden, moist, sunny/semi-shaded position. Mildly acidic to alkaline pH, grows well in heavy loams [11]. Species: *I. sibirica* (H7).
		Sino-Siberians 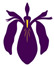	subg. *Limniris*, sec. *Limniris*, ser. *Sibericae*, subser. *Chrysographes* (includes hybrids)	Seepage zones and moist pastures, but not in shallow water. Distribution: Himalaya and South-West China [11].	Prefer damper, more boggy ground but not subject to prolonged flooding. Unsuitable for herbaceous borders [11]. Species: *I. chrysographes* (H6).
		Spurias 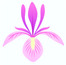 (some tolerate saline conditions)	subg. *Limniris*, sec. *Limniris*, ser. *Spuriae*	Ample moisture in soil, plenty of sunshine and no desiccation during summer. They grow in wet grasslands, marshes and river valleys, including saline conditions. Distribution: Europe, Asia and North Africa [11].	Prefer heavy clay loam soil, neutral to slightly acidic pH and adequate moisture [11]. Species: *I. graminea, I. spuria* subsp. *halophila*, *I. sintenisii*, (H6).
		Water irises 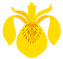 (invasive/cultivated aquatic macrophytes)	subg. *Limniris*, sec. *Limniris*, ser. *Leavigatae*	Wet, grassy places. Distribution: North America, Europe, Asia [11].	Moist to wet, deep substrate, humus-rich soil, acidic pH; suitable for the margins of ponds and streams [29]. Species: *I. pseudacorus* (H7), *I. ensata* (H6), *I. versicolor* (H7).
		Louisiana irises 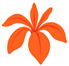	subg. *Limniris*, sec. *Limniris*, ser. *Hexagonae*	Grow in wetlands. Distribution: United States, mainly Louisiana but not exclusively [11].	Damp and humus-rich soil, areas with high temperatures in summer [29]. May be too tender for temperate climates [11]. Species: *I. fulva* (H5).
		Unguiculares 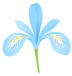 (Algerian iris is the best known)	subg. *Limniris*, sec. *Limniris*, ser. *Unguiculares*	Rocky/stony places, banks and dry scrub, open woodland. Distribution: Southern Europe, Middle East, North Africa [11].	Quick-draining soil with neutral to alkaline pH; full sun; suitable for the base of sunny walls [29]. Species: *I. unguicularis* (H5).
	Crest	Crested irises 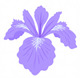	subg. *Limniris*, sec. *Lophiris*	Moist woodland. Distribution: North America and East Asia [11].	Moist humus-rich soil, in full sun/partial shade outdoors. In pots with loam-based compost with grit and leaf mold, moderate watering and moist during dormancy [29]. Species: *I. tectorum* (H7), *I. cristata* (H6), *I. japonica* (H4).
Tuberous roots		Himalayan irises 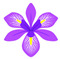	subg. *Nepalensis*	Distribution: Himalaya [11]	Substrate should be rich and moist from spring until autumn, with dormancy over winter. Species: *I. barbatula*, *I. decora* (H7).
Bulb		Reticulata 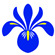 (highly cultivated garden bulb)	subg. *Hermodactyloides*	Open ground, mountain grasslands, stony slopes, sunny exposure. Distribution: Asia [11].	Preferably grown in open ground, raised beds, or bulb frames. Humidity should be ensured during growth [11]. Species: *I. reticulata* (H7), *I. histrioides* (H7).
		Juno irises 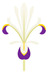 (appreciated for pots but very sensitive)	subg. *Scorpiris*	Semi-arid steppe and mountains, seasonally moist, on grassy slopes, receiving cold winters and hot dry summers. Distribution: mostly Asia with one exception [11].	Well-drained soil with neutral to slightly alkaline soil in full sun. Only in greenhouse/alpine house in deep containers with loam-based compost and grit, maintaining low humidity. Moderate watering [29]. Species: *I. nicolai*, *I. bucharica* (H5).
		Dutch, English and Spanish irises 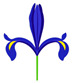 (cultivated for commercial cut flowers)	subg. *Xiphium*	Grow in soils that dry out in summer. Distribution: South-West Europe, North Africa [11].	Alkaline well-drained soil and dry over summer during dormancy [11]. Bulbs can be forced in large-scale production for cut flowers sold in flower shops [107]. Species: *I.* × *hollandica* (H5), *I. latifolia* (H7), *Iris xiphium* (H5).

Note: ^1^ An example of a recognizable flower type for the category and relevant short notes. ^2^ H indicates hardiness of species assigned according to RHS scale: H7—very hardy (colder than −20 °C); H6—hardy in very cold winter (−20, −15 °C); H5—hardy in cold winter (−15, −10 °C); H4—hardy in average winter (at most −10 °C). When all species mentioned in a category have the same hardiness, this is mentioned just once. It includes popular species examples for each category; the first example given in each category is depicted in the pictogram.

#### 5.4.2. Vegetative Propagation

Orris species (*I. florentina*, *I. germanica*, *I. pallida*) present rhizomes that produce one new rhizome section (called *struma*) per year of growth on average. The planting material is a section obtained from the first swelling of a harvested three-year-old rhizome that contains a minimum of three rootlets [108]. However, since the rhizome is the plant material sold for profit in these species, alternatives have been researched for their propagation [109]. *I. × hollandica* commercial bulbs are propagated through vegetative methods. Natural vegetative propagation is low, but the bulb chipping method can be successfully used [73].

#### 5.4.3. Micropropagation

In the industrial species *I. pallida*, somatic embryogenesis using flower buds collected at the beginning of floral stem development (immature buds) resulted in a shorter time to plantlet multiplication (only 7 months) compared to the use of young buds (side buds from completely developed flowering stems), which accounted for a longer duration until plantlet multiplication (10 months). However, there were differences in response to media between ecotypes. The iridal profile (iripallidal and iriflorental) in the rhizome resembled the profiles of mother plants, indicating that such a method preserves the quality required for planting material in industrial *Iris* crops and might save costs for farmers that use rhizomes to establish their crops, which could otherwise be sold for processing [109]. Furthermore, results indicate that artificial iris seeds can be obtained by encapsulating somatic embryos in sodium alginate beads, enabling the preservation and storage of stock material [110]. Research on *I. aphylla* showed that isolation of the embryo from the seed and in vitro propagation of the embryo were effective to circumvent the difficulty of propagation via seed germination [101]. In the bulbous *I. × hollandica* ‘Professor Blaauw’, an efficient in vitro propagation protocol was recently established using meta-topolin for the first time, which can enable large-scale production [111]. Optimized micropropagation methods for geophytes can be achieved by implementing the most novel approaches, such as improved bioreactor systems and Artificial Intelligence (AI) assistance [112]. A recent study demonstrated that cold plasma treatment of *I. reichenbachii* callus promoted meristematic activity and stimulated the production of specialized metabolites. The treatment had a lasting effect on the iris calli, enriching it with valuable compounds such as irisolidone and irilone [113]. This highlights the potential to obtain natural products that cannot be chemically synthesized.

### 5.5. Crop Management and Target Outcomes

#### 5.5.1. Field Crops: Industrial Irises

Field crops are established for industrial purposes (orris and other medicinal important species). The orris industry relies on the cultivation of certain iris species for their rhizomes: *I. florentina*, *I. germanica* and *I. pallida* [41,98,108]. The standard cultivation technology of industrial *Iris* recommends establishing the crop using rhizome segments of about 40 g in weight having 2–3 shoots/buds and roots of 2–3 cm in length. Planting is performed in October or in early spring at a density of 8 plants/m^2^ at distances of 50 cm between rows and 25 cm between plants in a row in furrows at 10 cm depth. To plant 1 ha of crop, 700–1000 kg of rhizome is used. The primary tasks involve weeding, disease and pest control and fertilization, the latter of which can be performed in autumn at a rate of 80 kg/ha P_5_O_5_ and in spring with 30 kg/ha N. In September of the second or third year, plants can be harvested. The yield is about 10–20 tons of fresh rhizomes/ha. These are cleaned and placed to dry, either whole or sliced. The yield for dry rhizomes is 2.5–3 tons/ha [98].

In Italy, with a long tradition of cultivating these plants, irises were grown as an intercrop in vineyards and olive orchards and established on narrow and steep plots of land for which mechanization is unsuitable. In these conditions, all work is usually performed by hand. Rhizome segments are planted in September at a distance of 30 cm between plants in furrows and 7–8 cm deep. In the summer of the third year, the plants are dug up entirely, and rhizomes are sold to the extraction companies as whole rhizomes, peeled or sliced [108]. Notable orris productions come from Morocco, with an estimated production of 120 tons of *I. germanica* annually; China, with about 100 tons of *I. pallida*; France, with about 40 tons of *I. pallida;* and Italy, once the hallmark of orris production but now limited to the areas of Chianti and Pratomagno in Tuscany, where less than 30 tons is produced annually. In Eastern Europe, several emerging countries have entered the orris market [8,108].

#### 5.5.2. Irises in the Landscape: Ornamentals

Irises are an emblematic presence in the landscape of Southern Europe, where they grow in dry places such as calcareous hills and coastal slopes [17]. These species gave rise to the most cultivated groups of irises [114]. Among these, the tall bearded group of tetraploid genotypes is now widely cultivated [115]. Wild irises contribute to the floristic diversity of natural landscapes. Whether wild, naturalized or cultivated in semi-natural gardens, *Iris* species can improve landscapes by fixing the substrate, contributing to the preservation of coastal zones and preventing land erosion [17].

The interest in irises from a gardening perspective remains extremely high, considered the “aristocrats” of gardens [115]. Hybridization creates new attractive cultivars with fascinating colors [116]; naturally occurring hybrids or ornamental plants of interest are still identified [114], and wild species are also introduced into cultivation [117]. The diversity of the genus ensures that there is an iris suitable for any garden type, ranging from water gardens (*I. ensata, I. pseudacorus*) and bog gardens (*I. sibirica*) to dry gardens (*I. pallida*) and rock gardens (miniature varieties). The TB, Spurias, Japanese and Siberian horticultural groups can be planted at the back of perennial borders or as a center island. Shorter varieties can be used to create large middle-ground masses. The bulbous cultivar groups of Dutch and English irises are suitable for front displays, whereas SDB species can be used for accents. MTB varieties are used for irregular patches in perennial borders. Most irises require full sun, but some are suitable for partial shade, such as *I. gracilipes* [11,115,118]. Phenological sequence of species can facilitate the extension of iris garden displays from early spring to summer. Some species can also exert an eco-function by supplying nectar to bees. Gardening sources recommending their integration and cultivation are helpful in landscaping with irises or pairing them with other ornamentals. Soil conditioning and amendments to improve pH can be applied according to the species needs [115].

#### 5.5.3. Constructed Wetlands

Water irises are the most used species for phytoremediation. By far the most commonly used for this purpose is *I. pseudacorus* [119]. This species thrives in moist conditions and can rapidly grow in natural wetlands (along water bodies and shorelines). After *I. pseudacorus* becomes established, the underground plant parts retain sediments and organic matter. The compact rhizome mat increases the rate of siltation and sedimentation, improving the suitability for water iris seedlings to grow in a positive feedback loop [120]. This phytoremediation technology relies on the basic principles of natural wetland ecosystems by creating constructed wetlands (CWs), which are artificial systems designed to treat wastewater. These CWs can be classified based on six criteria, among which flow pattern (horizontal or vertical), substrate (gravel, soil, or sand) and loading type (continuous or intermittent) are the most notable [121]. A recent study from Italy investigated the capabilities of a multistage CW system designed to treat combined sewer overflows of urban wastewater (a system still existing in Europe). The CW system was created to intercept part of a pollution load of 890,000 m^3^ per year and ensure compliance with discharge limits set in legislation. The CW presented two vertical subsurface flow beds (comprising filter medium layers of gradient-sized gravel) with separate hydraulic sectors planted with aquatic macrophytes and free surface water wetlands of varying depths planted with autochthonous aquatic macrophytes, including *I. pseudacorus*. The sampling showed that microplastic abundance correlated with flow rate and total suspended solids, with interesting prospects for dealing with the most pressing issues of modern sources of environmental pollution: microplastics in wastewater systems [122]. The phytoremediation applications of *I. pseudacorus* CWs can be further optimized through synergism with periphytic biofilm for enhanced efficiency in removing pharmaceutical contaminants from wastewater [123]. Furthermore, a study in a horizontal-subsurface-flow CW demonstrated that aerating the bottom of the first part of the filtering system planted with *I. sibirica* can enhance wastewater treatment capabilities for chemical oxygen demand, ammonium nitrogen and total nitrogen [124]. Therefore, additional studies on the factors that facilitate the successful mitigation of contaminants in CW systems are necessary to further optimize their performance and upscaling. Furthermore, a strong understanding of how *Iris* plants respond to pollutant stress could also prove important and deserves further attention.

#### 5.5.4. Protected Crops and Forcing Bulbs: The Cut Flower Crops

Dutch irises (*I.* × *hollandica*) are commonly found in flower shops and sold as cut flowers for bunches. The starting materials are bulbs that undergo treatment in controlled conditions to induce flowering, which is called forcing. This cultivation technology is well documented in floriculture [125]. The key to this technology is breaking the dormancy of the bulb. Dutch iris forcing requires a cold temperature duration of 6–13 weeks at 9–15 °C for bulb dormancy breaking. Ethylene or 30 °C pre-treatment might be needed [126]. The application of a 600 mg/L gibberellic acid treatment was shown to be beneficial for the bulb sprouting, flower parameters and vase life of cut flowers [127]. Studies indicated that temperature [128], bulb size and light were notable factors [129] in successfully forcing irises for cut flowers.

#### 5.5.5. Diseases and Pests

Ornamental iris cultivars are subject to fungal and bacterial infections, which can be worsened by less-than-optimal conditions for growth in planting locations (such as urban environments where conditions are more challenging) or by incorrect management [130].

**Fungi:** Commonly identified mycoses that produce symptoms on leaves decrease the aesthetic value of these plants in the landscape, such as rust (*Puccinia iridis*) and ink disease (*Bipolaris iridis*) [131], leaf spot (*Cladosporium iridis*) [130], black tip (*Didymellina poecilospora*) [132] and grey mold (*Botrytis cinereal*) [130]. Mycosis caused by *Botrytis convoluta* and *Athelia rolfsii* can affect rhizomes, and in these cases the plants are better dug up and removed entirely [131]. Species with bulbs can also be affected by fungi that develop during storage, such as the pathogens *Fusarium oxysporum*, *Sclerotinia sclerotiorum*, *Sclerotium tuliparum*, *Rhizoctonia* sp., *Rhizopus* sp. [132] and *Penicillium* sp. [133]. There have been new reports in recent years of iris diseases, suggesting the need for new measures in preventing the spreading of emergent pathogens. Anthracnose (*Colletotrichum truncatum*) was reported in *I. lactea* [134], and alternariosis *(Alternaria alternata*) was reported in *I. sanguinea* [135] in China. Foliage mycosis can be prevented or treated with fungicides such as chlorothalonil, mancozeb, myclobutanil, propiconazole, or triadimefon. Since iris leaves have thick waxy cuticles, adhesive has to be added to the treatment solution. It is important to remove diseased or dead leaves because they can act as inoculum sources for further infections [131]. Some treatments are recommended specifically for the bulbs [132].

**Bacteria:** Bacterial rot caused by *Pseudomonas* and *Erwinia* can develop from the base of leaves in bearded iris cultivars [130]. *Pseudomonas syringae* was reported recently in *I. foetidissima* from a garden center in Britain, with symptoms on its leaves [136]. *Xanthomonas tardicrescens* can affect the leaves, while *Pectobacterium carotovorum* inflicts the rhizome. One solution is to clean and rinse the rhizome with a low concentration of Cl (chlorine) solution followed by replanting. Alternatively, infected plants should be removed to prevent the spread of the infection to other plants [131].

**Viruses:** Iris severe mosaic virus (ISMV, Potyviridae) causes leaf stripes and mosaic patterns on the leaves of several species of *Iris*, including bulbous species [137]. This infection has a negative impact on the production and marketability and cannot be treated. Therefore, early detection in the leaves and rhizome based on targeting the highly conserved 3′ untranslated region of ISMV was proposed as a precision approach in preventing the planting of infected stock for commercial crops [138]. Iris mild mosaic virus (IMMV, Potyviridae) can also affect irises, causing yellow streaks on the leaves, mottling and flower breaking [132]. Iris yellow spot virus (IYSV, Bunyaviridae) can be transmitted by *Thrips tabaci* (order: Thysanoptera) and was reported in *Iris × hollandica,* yet it can infect a wide range of other bulbous species and has become widespread [139].

**Pests:** The most impactful iris pests are *Myzus persicae* (order: Hemiptera), which affects the foliage; *Macronoctua onusta* (order: Lepidoptera), which damages the rhizome; *Ceutorhynchus* sp. (order: Coleoptera), which damages the seed capsule; and *Gryllotalpa gryllotalpa* (order: Orthoptera), causing damage to young shoots in spring. Dimethoate treatments can work well for these pests and should be applied outside of the flowering period. Furthermore, some rodents feed on the rhizomes [131]. The larvae of the fly species *Neorthacheta dissimilis* (order: Diptera) can infest *Iris* flowers, causing aesthetic damage. One study showed a high susceptibility of the common ornamental taxa to this pest, hinting to wider issues it might yet cause [140]. The underground plant parts are also susceptible to nematodes (phylum: Nematoda): *Ditylenchus iridis*, *Meloidogyne incognita* and *M. ichinohei* [132]. Florivores can also damage the flowers [141].

Across several varieties of bearded cultivars, Seraya et al. [130] identified diverse fungal complexes in both the rhizosphere and phylloplane in the urban conditions of Moscow, concluding that the ornamental value of plants was reduced the most by mycoses, followed by bacteriosis and lastly by nutrient-deficit soil [130]. Chloropicrin has been reported as an effective soil disinfectant before planting, protecting against a large spectrum of diseases and pests, along with other substances [132]. Other gardening sources report benefits from dusting with wood ash [115].

### 5.6. Invasive Species Management

*I. pseudacorus* is a prolific wetland species that can invade and dominate a wide range of vegetation types, reducing diversity and successional trajectories by colonizing habitats and giving rise to monospecific stands that outcompete other plants, including marsh vegetation from the genera *Typha*, *Carex*, *Schoenoplectus* and *Equisetum,* among others [120]. Issues have been reported in North America, Japan, China, Argentina, South Africa and Australasia [142]. The effectiveness of mechanical control is somewhat limited because small rhizome fragments can be dislodged and flow downstream, where they can become established. Mowing the flowers before they produce seeds may slow the spread, while installing benthic barriers can restrict the speed of rhizome spread. Chemical methods involve applying herbicides such as glyphosate at rates of 5% to 8% between spring and early summer and 2% to 8% in autumn. Boom-sprayer application with both glyphosate and imazapyr, as well as drizzle application of imazapyr, was shown to be effective [120]. Additional biological control agents are also being assessed for potential applications [143] and may soon be available.

## 6. Phytochemistry of *Iris* spp.

### 6.1. Most Valuable Iris Compounds

Recent studies on the phytochemistry or *Iris* spp. have highlighted their importance as a source of valuable compounds [144]. In the genus *Iris*, phenolic acids, an abundance of flavonoids, small amounts of alkaloids, primary metabolites and essential oils have been identified [2]. *Iris* leaves are a rich source of phenolic acids such as ferulic, p-coumaric, sinapic, coffeic, chlorogenic, neochlorogenic, p-hydroxybenzoic and vanillic acids [89]. The most important flavonoids found in *Iris* leaves and flowers are C-glycosides, with two flavonols (kaempferol and quercetin) frequently occurring, but these are not limited to the genus, also being found in other plants of the Iridaceae family. By contrast, several isoflavones such as irigenin, tectorigenin and irisolone have only been reported in the *Iris* genus [27]. Isoflavonoids that contain a methylenedioxy group in ring A (such as irilon, irisolidone, nigricin, tectoridin, etc.) are rather rare in the plant kingdom. In this regard, the species of the family Iridaceae can represent a valuable source of such compounds [89]. In support of this rediscovered importance, over 80 such compounds have been isolated and described for this group [144]. New compounds continue to be isolated from species of this genus [145], and species that have not been comprehensively characterized are now attracting scientific interest [3]. Novel approaches in metabolic profiling are also instrumental for precise phytochemical characterization. A study on *I. versicolor* used high-resolution mass spectroscopy and in silico fragmentation for highly reliable identification of compounds [146].

Irone is the name given to a mixture of C14 monocyclic ketones obtained from the rhizomes of some *Iris* species, namely *I. florentina*, *I. germanica* and *I. pallida*. Irone is derived from triterpene precursors named iridals during the aging of peeled and/or sliced rhizomes [8]. This is the most valuable product obtained from iris crops, with a violet scent that is used primarily in perfumery [41]. To date, five geometrical irone isomers have been fully identified in *Iris* (Table 2): *cis*-α-irone, *trans*-α-irone, *cis*-γ-irone, *trans*-γ-irone and β-irone. The latter is thought to be a product of one of the former two during rhizome processing. Either can be found as (+) and (−) enantiomers, depending mostly on species [8], and they differ in scent [147]. Dextrorotatory irones are found in *I. pallida,* whereas levorotatory irones are found in *I. germanica* [147,148]. Although structurally similar, α-/β-/γ-irones differ in the position of the double bond on the cyclohexane ring [149]. Because only α- and γ-irones are naturally occurring compounds, they can be referred to as markers of orris quality.

### 6.2. Influencing Factors

Among the factors influencing the phytochemistry of these plants, a study on *I. variegata* showed that genetics and environment (light regime) together influence the leaf phenolic profile [153]. The cultivation technology has a great impact on the quality of the product obtained. A study demonstrated the sensitivity of some *Iris* cultivars to mineral nutrition in regard to phenylpropanoid metabolism, and the authors further concluded that it can predict cultivation with increased content of bioactive compounds for medicinal crops [154]. A comprehensive experiment in Tuscany (Italy) investigated the influence of plant density and harvesting age on *I. pallida*. The results indicated that density significantly influenced the biomass variables and orris essential oil yield, with lower values being more favorable [155]. This suggests that further optimization of cultivation technology can ensure a superior yield and quality of metabolites of commercial interest.

### 6.3. Harvest and Post-Harvest Matters: Aging Rhizomes and Processing

From planting to extraction, orris crops undergo at least a six-year cycle: three until rhizomes are harvested in the summer and approximately another three for aging the orris [108]. To obtain black-type orris, harvested rhizomes are washed, sliced and dried, and for white-type orris, they are peeled by hand and undergo slow drying for a few years [156]. The drying of rhizomes begins by spreading the material in one layer in the sun before being placed in storage [157]. This operation can also be performed artificially at 30–35 °C as an alternative to natural drying. The drying rate is about 4:1 [98]. The drying method has an influence on the phytochemical composition of the *I. germanica* rhizomes, with encouraging results for microwave drying that enhanced the accumulation of irone content. This could be adopted as a viable post-harvesting processing technique [158]. Traditional aging of rhizomes is a slow desiccation process in which oxidative degradation of iridals leads to the slow accumulation of irones. This is a natural process and results in a natural product of high value [108,155]. For example, the essential oil of *I. florentina* rhizomes from Turkey aged for only 3 months had merely 4% α-irone and 8% γ-irone [159], while essential oil from *I. germanica* rhizomes, also from Turkey, aged for around a year and a half to two years had 30–31% α-irone and 55–59% γ-irone content [160]. Furthermore, the irone isomers present are indicative of the species. Essential oil from *I. germanica* rhizomes from Morocco aged for two years had 62% *cis*-α-irone, 37% *cis*-γ-irone and 1% *trans*-α-irone, while *I. pallida* essential oil had 61% *cis*-γ-irone, 34% *cis*-α-irone and 5% *trans*-α-irone [161]. The different major isomers between the two species are notable and have been consistently observed and reported [147].

It is possible to accelerate the artificial aging of rhizomes to produce irones by using patented methods via various chemical oxidation processes (e.g., H_2_O_2_, KMnO_4_, sodium/ammonium nitrite), ionizing radiation, O_2_/lipoxygenase, pressurized oxygen, thermal incubation (50 °C) and bacteria and fungi, to name a few [8]. However, there remains a preference for the naturally obtained product, considering the luxury options of the cosmetic and perfumery industry as well as the food industry, which are driven by customer preferences for natural botanicals. According to industry specification, dry grounded rhizome is steam-distilled to obtain orris essential oil, also known as orris butter or orris concrete (yield of 0.2–0.3%), with an irone content of 13–17% and a high amount of partially esterified fatty acids (>80%), which causes the product to be a solid mass at room temperature and yellow to brown in color. The melting point of orris essential oil is 40–50 °C. The alcoholic separation of fatty acids results in orris absolute with a high irone content of 55–85%. The orris resinoid is obtained by benzene extraction or using petroleum ether from comminuted dried rhizomes; *cis*-γ-irone and *cis*-α-irone are the compounds responsible for the violet-like scent [162]. The prices are high considering the long process required to obtain orris essential oil, costing several thousands of euros per kg [155], and for this reason, research has also been directed towards synthesizing them in the lab [149].

For medicinal purposes, *Iris* leaves from bearded species and cultivars can be harvested from May to October for their phenolic compounds, such as hydroxycinnamic acids, xanthones, flavonoids and isoflavonoids, in addition to other phytoactive compounds [89]. Efficient and optimized isolation and extraction methods are also being researched. A study on *I. sibirica* indicates that deep eutectic solvents obtained by mixing choline chloride or tetrabutylammonium bromide with various hydrogen bond donors enable the selective recovery of bioactive compounds [163].

### 6.4. Quality Standards for Orris and Medicinal Irises

The purpose of quality standards in the medicinal *Iris* chain is to promote and implement practices that ensure a reliable quality of medicinal raw materials (in regard to composition and bioactivity) that have a reduced environmental impact and can meet the specific standards of the pharmaceutical industry [89].

The latest “Good Agricultural and Collection Practice (GACP) for Starting Materials of Herbal Origin” provides updated guidelines to ensure the high quality of medicinal products [164]. For plant material collected from irises that is destined for processing for medicinal purposes, the collection and cultivation should also adhere to these practices. The use of pesticides and herbicides should be avoided, and fertilizers should be used at the minimum level in accordance with these guidelines. Furthermore, farm certification schemes [165] could also be implemented for *Iris* crops.

The quality-by-design approach (QbD) regards pharmaceutical products and places importance on building their quality via quality control at each technological stage, as devised for other important medicinal geophyte species such as *Panax notoginseng* [166] or *Crocus sativus* [167]. This could also be implemented for the medicinal *Iris* chain, as the potential of these plants extends into pharmaceutical applications. The pharmacognostic standardization of medicinal *Iris* is necessary to ensure consistency in therapeutic applications, as has been laid out for *I. germanica* [6]. However, there are many other medicinal irises for which such standardization is not currently available.

Regarding the reception of dry rhizome material, called orris (*Rhizoma Iridis*), the current technical standards consider 5% rhizomes (at most) with brownish interiors and 2% rhizomes with left roots as impurities [98]. International Organization for Standardization (ISO) 18054:2004 regards oils of orris rhizomes obtained from *I. pallida* or *I. germanica* and provides the standard procedure for the determination of irone content [168]. In the European Pharmacopoeia (Ph. Eur. 10), *I. domestica* is the only species from the genus *Iris* included among the official monographs [169]. Orris extract destined for use in the food industry as flavors in the United States has been assigned GRAS (Generally Recognized As Safe) numbers 2829 and 2830 [170]. Orris extracts can be found in products that feature a WONF notice (With Other Natural Flavors) on their label [8]. More standards are expected to be put into place as the range of applications expands.

## 7. Overview of Multipurpose Applications

### 7.1. Summary of Historical and Established Use

Plants of the genus *Iris* have been known and used by various populations since ancient times, with written historical attestation from ancient authors such as Pedanius Dioscorides [171] and Pliny the Elder [172]. Their main historical applications have been summarized in a previous work [41], but a brief account of the notable historical uses of various plant parts is given here. Irises possess documented tinctorial properties. The blue and green colors derived from their flowers were used by medieval manuscript illuminators in Europe [173]. Black paint was also obtained from crushed *I. pseudacorus* rhizomes mixed with ferrous sulfate [174]. Its application in food is also not new; iris rhizome has been used as a spice in oriental cuisine, and to this day it is used to flavor various foodstuffs [175] such as in the gin and vermouth industry in Italy [8]. Iris has been used as a freshener since the Late Medieval period and the early Renaissance. Rhizome powder was spread on linen ruffs in storage by the English, and pieces were boiled with linen by the French. Fine large rhizome pieces were turned into ornamental or useful items [41]. The leaves of *I. tenax* were used to obtain fiber for use in fishing nets and snares by Native Americans [27]. There is evidence of its medicinal use across all main cultures where irises grow [2]. *I. versicolor* was among the most valued Native American medicinal plants [27]. *I. bungei* has been used in traditional medicine in China and Mongolia [176]. Avicenna recommended the use of *Iris* rhizome for various external applications [174]. In Europe and the United States of America, early botanists, herbalists and medicinal reference works (such as *A Supplement to the Pharmacopoeia* by Frederick G., S. or *American Materia Medica* by Ellingwood, F.) mention some well-established properties and medicinal uses of various irises [41]. While ethnopharmacological uses have been identified for about 25 *Iris* species in a recent review [2], there are most likely many more species used in traditional medicine, as the information is not yet fully centralized. From ancient times until today, perfumery has been a well-established use [171]. Processing innovations, such as the mechanical grinding of orris, have been reported from the XIXth century, an era of great development for the fragrance industry which made them more available and affordable [177].

### 7.2. Novel Evidence on Biologic Activity

#### 7.2.1. Antimicrobial and Antioxidant Activities

The antibacterial, antifungal, antiviral and antioxidant activities of iris extracts and compounds play defensive roles against microorganisms and free radicals.

There is increased interest in using plant-based extracts in mouthwashes against oral bacterial biofilms. The sub-Minimum Inhibitory Concentration (MIC) methanolic extracts of five *Iris* species (*I. germanica*, *I. lactea*, *I. pallida*, *I. versicolor*) rich in (iso)flavonoids were tested for anti-adhesion and antibiofilm potential, relevant in limiting dental plaque. Results showed that *I. pallida* leaf extract was the most effective. A phytosterol derivative (7-β-hydroxystigmast-4-en-3-one) was correlated with quorum sensing inhibition [178]. These strongly suggest a worthwhile path of further studies regarding the applications of *Iris* extracts for oral health. Another great concern in medicine is the rising incidence of resistant strains. A study on three *Iris* species (*I. pumila*, *I. reichenbachii* and *I. illyrica*) successfully tested crude extracts against the multi-resistant bacterial strain methicillin-resistant *Staphylococcus aureus* subsp. *aureus* ATCC 33591 (MRSA). Results suggested that isorhamnetin from *Iris* spp. had high binding affinity to the SauPBP2a active site, representing the protein responsible for the bacterium’s resistance to β-lactams [179]. *I. confusa* is a species with known medicinal properties used in the treatment of bacterial infections and gastritis. A study screened this plant’s potential against *Helicobacter pylori* strain ATCC 700392, a bacterium associated with chronic gastritis and duodenal ulcers and a risk factor for gastric cancers. The crude polar fraction of underground plant parts inhibited *H. pylori* with an MIC value of 62.50 μg/mL. Furthermore, some isolated compounds were shown to be even more effective alone, such as irigenin [180]. *I. tenuifolia* has been used in Mongolian traditional medicine for kidney disorders. Ethanol extracts of underground plant parts revealed new chromane derivatives. The newly identified compounds were tested against eight microorganisms. Of these, a new cycloflavan (5-methoxy-6,7-methylenedioxy-4-O-2′-cycloflavan) showed promising activity against *Bacillus subtilis*, *Enterococcus faecalis* and *Mycobacterium vaccae* [145]. Aqueous and methanolic extracts of aerial plant parts of *I. persica* subsp. *persica* were tested for their antimicrobial potential. Shikimic acid was one of the most abundant compounds in both extracts, yet the aqueous extract was slightly more effective against microorganisms than the methanolic one, most likely due to the presence of several minor phenolic compounds (including hesperidin) detected only in the aqueous extract. Overall, the best inhibitory effects of the extracts were against *Klebsiella pneumoniae*, *Staphylococcus aureus* and *Candida parapsilosis* [181]. *Acanthamoeba* keratitis is a serious ocular infection caused by certain opportunistic protozoa. An extract of whole *I. setosa* plants was screened for its amoebicidal effect against trophozoites of *A. castellanii* and *A. polyphaga*, showing morphological and viability changes in a dose-dependent manner when exposed to the extract, while having low impact on HCE-2 (human corneal epithelium cells) [182].

In addition to significant human pathogens that are of increased focus, plant viruses that are particularly difficult to deal with have devastating economic consequences in crop plants. Dutch iris (*I.* × *hollandica*) bulbs express two types of ribosome-inactivating proteins (IRIP and IRA) that have proven antiviral activity against viruses such as Tobacco mosaic virus (TMV) and Tobacco etch virus (TEV). Such plant-derived proteins could be used for the management of these diseases in major crops; this is worth exploring further, as their mechanisms of action are yet to be fully explained [183].

Oxidants and radicals remain major drivers of degenerative processes that can favor inflammatory processes, increasing the risk and progression of chronic illnesses [184] and aging [185]. Therefore, antioxidants remain one of the most intensively studied aspects of biological activity amongst plant compounds [186]. An investigation into the antioxidant properties of polar extracts of the aerial parts and rhizomes of *I. postii* revealed that a total methanol extract of the aerial parts and an n-butanol sub-extract of the methanolic extract obtained from rhizomes displayed high total antioxidant activity [187]. Methanolic extracts of *I. pseudacorus* aerial parts and rhizomes were tested via a 2,2-difenil-1-picrilhidrazil (DPPH) assay. The rhizome extract at a concentration of 125 μg/mL showed the highest radical scavenging activity, with 60.75–75.84% inhibition [188]. *I. kashmiriana* polar flower extracts (with ethyl acetate and methanol) scavenged DPPH free radicals following a dose-dependent trend. The methanolic flower extract demonstrated a maximum inhibition of over 80% at a concentration of 800 μg/mL [189]. Exosomes derived from *I. germanica* rhizomes significantly decreased H_2_O_2_-induced reactive oxygen species (ROS) damage in nHEKs (human epidermal keratinocytes), therefore demonstrating a protective effect on skin that might be developed into products for anti-aging and skin conditions [190].

#### 7.2.2. Pharmacological and Immunomodulatory Potential

The pharmacological and immunomodulatory effects of iris extract and compounds, as systemic biological responses, are often screened and studied in vitro on cell models or in vivo on model lab animals. Therapeutic or pharmacokinetic observations are important for moving forward in deploying effective plant-derived drugs. Anti-inflammatory, immunomodulatory, cytotoxic and antiproliferative activities are of particular interest.

One study showed that irigenin isolated from the underground plant part of *I. confusa* was a potent inhibitor of cyclooxygenase-2 (IC_50_ of 10.83 μM), an enzyme with a role in mediating inflammation, therefore presenting the possibility to be used in mitigating inflammation [180]. An extract from the aerial part of *I. florentina* [*I. albicans*] indicated an anti-nociceptive effect on mice that might suggest its potential use as an analgesic [191].

A rhizome extract of *I. pseudaorus* was tested on MCF-7 (breast cancer), HeLa (cervical cancer) and HCT-116 (colorectal carcinoma), with results indicating higher cytotoxic activity against MCF-7, with an IC_50_ of 11.75 μg/mL [192]. A newly isolated oligostilbene from *I. lactea* seeds at 100 μM reduced the viability of HepG2 (hepatoblastoma) and PC12 (pheochromocytoma) [193]. A new isoflavone-type compound from *I. tenuifolia* (5,2′,3′-trihydroxy-6,7-dimethoxyisoflavone) demonstrated potent activity (GI_50_ = 7.6 µM) against the K-562 cell line (human leukemia cell line). This antiproliferative inhibition activity was attributed to the ortho-dihydroxyl groups in the B-ring structure of the molecule [145]. *I. barnumiae* methanolic extracts from flowers and rhizomes rich in quinic acid were tested for antiproliferative activity on cell lines PC3 (metastatic prostatic adenocarcinoma), MCF-7 (Michigan Cancer Foundation cell clone 7), U-87 MG (Uppsala 87 Malignant Glioma) and HT-29 (human colorectal adenocarcinoma cell line). The results indicated that the rhizome extract had a stronger antiproliferative effect but was more effective on HT-29 than on other cell lines [194].

### 7.3. Remediation Applications and Outcomes

The use of plants as main components in the mitigation of pollution is increasingly popular. The green technology of phytoremediation particularly focuses on the remediation of metals and metalloids [195]. Heavy metals are health-threatening pollutants that accumulate in the environment due to anthropic activities. Several *Iris* species have been shown to possess the ability to remediate heavy metals from soil or water, such as *I. ensata*, *I. germanica*, *I. halophila*, *I. lactea*, *I. latifolia*, *I. pseudacorus*, *I. sibirica*, *I. tectorum* and *I. wilsonii*. However, their tolerance to heavy metal toxicity varies. As perennial plants, irises are highly relevant for phytoremediation as a slow, long-lasting process. Once established, these plants can remediate essential metals that are toxic in excess, such as Cu, Fe, Mn and Zn, as well as non-essential metals for plants, such as As, Cd, Cr, Pb and Hg, which are notoriously difficult to deal with and highly toxic to organisms. The common mechanism in many of these species is phytostabilization [10]. In an experimental setting, *I. pseudacorus* plants were subjected to a solution enriched with 0.5–4.0 mg/L Zn and showed accumulation of Zn in their roots. The authors reported visible signs of toxicity symptoms in plants [196]. Mitigating the negative effects of the pollutant is important in optimizing phytoremediation. In this regard, it was shown that root colonization by arbuscular mycorrhizal fungi can be beneficial for alleviating stress, as shown in *I. tectorum* interaction with Cr [197] and As [37]. *I. pseudacorus* was tested for its remediation capacity when subjected to a mixture of metals, simulating a typical mine drainage. Plants were grown in synthetic wastewater with over ten heavy metals. The accumulation of heavy metals (Cd, Cu, Fe, Mn, Pb and Zn) increased during the 14 months of the experiment, most notably in the underground parts, which were determined to be the main plant parts responsible for phytoextraction. The average removal rates were high for Cd, Fe and Zn. The amounts of toxic metals in the biomass after a year were as follows: Cd: 1.15 mg; Cu: 10.88 mg; Fe: 1133.61 mg; Mn: 22.59 mg; Pb: 1.76 mg; and Zn: 329.76 mg reported per kg [198].

Heavy metals are not the only pollutants of current concern. A comparative study on three wetland plants (*I. pseudacorus*, *Lythrum salicaria*, *Phragmites australis*) in a semi-hydroponic experiment screened the bioremediation capacity for a mixture of 27 frequent pollutants at concentrations of 1–5 µg/L, ranging widely from pharmaceuticals to pesticides. The results indicated the superior bioremediation capacity of *Iris* plants compared to the other two species, exceeding 80% removal efficiency for 22 of these pollutants within a month [199].

In addition to direct phytoremediation, irises can assist in obtaining materials further used for remediation processes; here, rather than living plants, extracts or processed plant biomass is utilized. Dye pollution is a major concern, as many dyes can have negative impacts on aquatic environments. Among these, Congo red is widely used. Fe_3_O_4_ nanoparticles were obtained from ferric chloride and n-butanol extract from the roots of *I. barnumiae*. The coated nanoparticles were successfully used as an adsorbent for Congo red removal from aqueous solution, demonstrating an adsorption capacity of 50 mg/g [194]. A study on *I. sibirica* showed that Cd removal efficiency by chemisorption was highest for stem biochar (19.92 mg/g), followed closely by leaf biochar (19.89 mg/g) and lastly by biochar from underground plant parts (13.22 mg/g). The study determined that stem biochar had a superior ion exchange and precipitation capacity, while root–rhizome biochar might be better for treating acidic wastewater [200].

### 7.4. Novel Niches

Weed control occupies a prominent role in cultivation technology, but concern for the ecotoxicity of herbicides encourages the search for allelochemicals. Seed extracts of *I. sanguinea* were shown to contain chemicals that can inhibit the germination of other plants [201]. β-ionone isolated from *I. pallida* (found in a concentration of 20 mg/g of rhizome) has also demonstrated promising allelopathic properties against several plants, with potential use as a bioherbicide [202], an application that might be explored more in the future.

In addition to their useful chemicals, *Iris* plants are natural hosts of a rich microbiome that, only in recent years, has been studied for its usefulness, with promising potential for new applications. Recently, the diversity of cultivable endophytic bacteria from the leaves and roots of naturally grown *I. pseudacorus* was screened, in which four isolates of the species *Bacillus toyonensis*, *Brevibacterium frigoritolerans*, *Pseudomonas gessardii* and *Streptomyces atratus* were identified, with antimicrobial activity against the most common plant pathogens from the genera *Alternaria*, *Botrytis*, *Fusarium*, *Pythium* and *Rhizoctonia*. Furthermore, 13 isolates were shown to produce indole-3-acetic acid (IAA), which can stimulate root and shoot growth in plants [203]. Six *Actinobacteria* strains were isolated from the roots, stems and leaves of *I. persica*. While this was a preliminary study, there is newly identified potential to apply such microbial supernatants as seed treatment for crop plants (e.g., wheat) to enhance growth and mitigate stress in seedlings [204]. However, further studies are needed on isolate strains from *Iris* plants to test their potential for producing microbial products.

## 8. The Past, Present Future and Interconnections Between Domains

**Figure 5:**
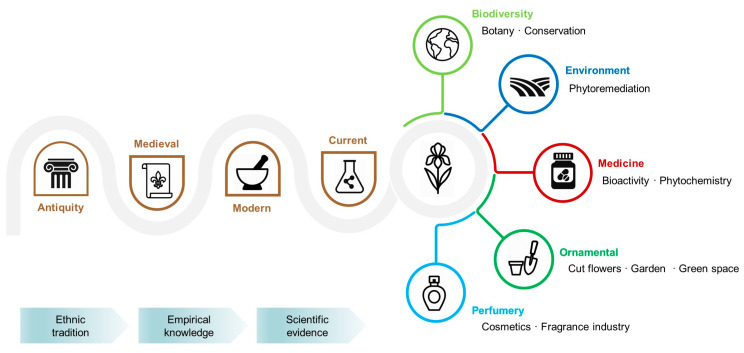
Temporal continuity of *Iris* from past knowledge to modern interest and current applications (original infographic by I.C.).

There is an undeniable continuity in the knowledge and use of *Iris* plants from ancient times to the present day. This knowledge underwent an evolution to reach the diversified field of applications of the present day (Figure 5).

Many species studied for the bioactivity of their compounds are recognized in traditional medicinal systems, while the orris industry has relied on the same species since ancient times. This positions the *Iris* genus as a perfect example of ethnobotanical continuity, where ancient recognition of its value informs and sustains its current scientific interest and industrial applications.

In retrospect of the examined literature, one can summarize that the iris flower remains a strong and recognized symbol. Its cultivation retains a long-standing tradition in Europe, especially in Italy, and holds strong cultural importance. On every continent where *Iris* plants grow, there are associated traditional uses. In the modern sense, the orris industry and ornamental potential of irises have been explored in more depth than others. However, in addition to these, the most impactful applications today are related to the bioactivities (antimicrobial, antioxidant, immunomodulatory and cytotoxic effects) of their compounds. The phytoremediation of toxic substances from the environment (such as heavy metals) is one such example of their contemporary importance. Novel niches explore their little-known microbiomes and seek to identify emerging applications in the near future.

Species cultivated with well-known technology (*I. florentina*, *I. germanica, I. pallida*) provide a highly advantageous steady and predictable supply of standardized quality plant material for the processing industry that supports perfumery, cosmetics and medicinal applications. Industrial and ornamental irises can be easily grown, since planting material is available for sale and most crop management needs are known, representing a safe investment. In addition to these, *I. pseudacorus* used in phytoremediation remains the best-studied plant for this purpose.

One may notice a change in the trend in the recent literature on this botanic genus. The older literature focused on a few species in regard to their industrial (orris), medicinal, or ornamental potential. However, recent research indicates a shift in interest that extends into biodiversity conservation and the discovery of new bioactive compounds. There is also a noticeable interest in phytoremediation and the use of *Iris* in biotechnology. To better make use of their demonstrated potential, expanding the range of cultivated *Iris* species may be necessary. This could be beneficial by firstly helping to preserve their genetic diversity but also by reducing the pressure on wild populations. This is especially important for endemic species that are at risk of becoming endangered. Secondly, from an agronomic standpoint, diversifying the range of cultivated species can foster the discovery of new desired traits, such as resistance to certain pests or diseases, unique ornamental qualities, useful microbiomes and new phytochemicals of interest.

A possible hindrance to fully benefiting from this potential resides in what is unknown regarding scaling up the cultivation of these lesser-known *Iris* species, because tailored cultivation technologies are needed first. Identifying the potential of newly studied species must eventually transition to successfully integrating them into agricultural systems for a steady, standardized supply of raw material. Therefore, the next steps would be to conduct more research on their specific growing conditions, pest management and post-harvest handling (including processing and extraction). There remains the possibility that not every species that shows potential for medicinal or other uses will necessarily become a good candidate for large-scale cultivation. Some taxa have specific environmental needs that could make them too challenging or costly to grow outside of their natural habitats, so their cultivation might not be feasible or sustainable. In such cases, it might be more practical to focus on in vitro propagation or tissue culture to extract valuable compounds. Therefore, defining the best approaches in accordance with each species of interest requires a balance between potential benefits and practicality and sustainability, ensuring that the efforts are both ecologically and economically sound.

Given that the recent literature brings evidence of new application niches for wild irises, such as unexplored microbiomes and phytochemistry, it is necessary to consider that biodiversity conservation can also support this as-yet-undiscovered potential for future applications. Wild *Iris* species support local ecosystems (e.g., various pollinators) and remain useful genetic repositories for breeding ornamental irises, a huge industry which extends worldwide. In addition, novel insights demonstrate that previously less-studied irises, specific plant properties, certain isolated phytochemicals and understudied microbiomes hold real potential for impactful applications in various domains. Based on the specific resources consulted, the following challenges were identified as requiring future attention and research:

- optimizing cultivation technologies to enhance the phytochemicals of interest and feasibility of *Iris* cultivation;
- perfecting phytoremediation systems and preparing them for scaling-up;
- breeding ornamental irises able to withstand stresses, pests and diseases for low-maintenance green spaces;
- devising cultivation technologies for newly identified useful species;
- pharmacognostic standardization for more species for safe pharmaceutical use;
- biodiversity conservation of declining wild populations and genetic studies (genetic diversity mapping of the existing wild populations and establishing ex situ collections);
- effective management approaches for invasive irises.

## 9. Conclusions

The multipurpose value of the genus *Iris* resides in its diverse importance, including medicinal plants (over 20 species), landscaping ornamentals (e.g., *I. ensata*, *I. germanica*, *I. pumila*) and cut flowers (*I.* × *hollandica*), as well as the production of orris essential oil for perfumery (*I. florentina*, *I. germanica*, *I. pallida*) and the remediation of environmental contaminants (*I. pseudacorus*). The domains of interest further diverge into novel niches. This in-depth literature study indicates a strong interconnectedness of past, present and future interest in the *Iris* genus. Ethnobotany studies can indicate unexplored useful species and inspire novel applications. New compounds with pharmacological and immunomodulatory potential have been isolated and studied. An examination of the literature on *Iris* fully supports the medicinal, ecological and industrial relevance of this genus in the current context.

## Figures and Tables

**Table 2:** Irones and their olfactory characteristics for perfumery.

Irone Isomers	Origin	Prevalence in Orris	Compound Scent
*cis*-α-irone	natural [8]	major constituent [149,150,151]	floral, green, light, [150], fine iris scent [148], woody and fruity [147]
*trans*-α-irone	natural [8]	minor constituent [149]	weak scent [149], sweet, dry, violet, rosy [147]
*cis*-γ-irone	natural [8]	major constituent [149,150]	floral, sweet, green [150], strong powdery [149], woody, dry, rosy [147]
*trans*-γ-irone	possibly occurs by isomerization from *trans*-α-irone [148], most likely an artifact from processing/aging	minor constituent [148]	soft orris butter, weak [148], woody [152], chemical-like [147]
β-irone	not biosynthetic, an artifact occurring from processing/aging [8]	minor constituent [149]	strong transparent fruity/green top note, rich violet floral middle note, lasting powdery and woody note [149]

## Data Availability

No new data were created or analyzed in this study. Data sharing is not applicable to this article.

## References

[1] Plants of the World Online|Kew Science *Iris* Tourn. Ex L.. https://powo.science.kew.org/taxon/urn:lsid:ipni.org:names:326330-2.

[2] Khatib S., Faraloni C., Bouissane L. (2022). Exploring the Use of Iris Species: Antioxidant Properties, Phytochemistry, Medicinal and Industrial Applications. Antioxidants.

[3] Friščić M., Maleš Ž., Maleš I., Duka I., Radonić A., Mitić B., Hruševar D., Jurić S., Jerković I. (2024). Gas Chromatography–Mass Spectrometry Analysis of Volatile Organic Compounds from Three Endemic *Iris* Taxa: Headspace Solid-Phase Microextraction vs. Hydrodistillation. Molecules.

[4] Davis C.C., Choisy P. (2024). Medicinal Plants Meet Modern Biodiversity Science. Curr. Biol..

[5] Daley D.K., Badal S., McCreath S.B., Clement Y.N. (2024). Chapter 5—Plant Crude Drugs. Pharmacognosy.

[6] Aara I., Faheem M., Shahista S. (2025). Pharmacognostic Standardization, Phytochemical Evaluation Potential of *Iris germanica* L.. World J. Intern. Med. Surg..

[7] Asgari E., Wani M.A., Al-Khayri J.M., Jain S.M. (2025). The Intersection of Biotechnology and Iris Breeding: A New Era in Floriculture. Breeding of Ornamental Crops: Bulbous Flowers.

[8] Bicchi C., Joulain D. (2025). A Comprehensive Review on Essential Oils and Extracts from *Iris* Rhizomes. Phytochem. Rev..

[9] (2025). Orris Butter Pallida (20% Irones) from hermitageoils.com.

[10] Naing A.H., Park D.Y., Park H.C., Kim C.K. (2023). Removal of Heavy Metals Using Iris Species: A Potential Approach for Reclamation of Heavy Metal-Polluted Sites and Environmental Beautification. Environ. Sci. Pollut. Res..

[11] White B., Bowley M., Brearley C., Christiansen H., Cohen O., Davis A., Dickson-Cohen V., Ellis J., Grey-Wilson C., Innes C. (2012). A Guide to Species Irises: Their Identification and Cultivation.

[12] Yu X.-F., Feng Y., Yue L.-J. (2021). Collection and Evaluation of Iris Species in Southwest China. Acta Sci. Pol. Hortorum Cultus.

[13] Linnaeus C. (1753). Species Plantarum.

[14] Robu T. (2005). Monograph of the Genus Iris: Physiology, Botany and Uses.

[15] Cristea V. (2014). Plante Vasculare: Diversitate, Sistematică, Ecologie Și Importanță.

[16] Kandeler R., Ullrich W.R. (2009). Symbolism of Plants: Examples from European-Mediterranean Culture Presented with Biology and History of Art: APRIL: Iris. J. Exp. Bot..

[17] Pignatti S., Savoia A.U., Piazza S.V. (2000). Iris: A Significant Element of the Mediterranean Landscape. Ann. Bot..

[18] Radenković L. (2013). Perunika—Cvet Nebeskog Ili Htonskog Sveta? German Iris—The Flower from the Heavenly or Chthonian World?. Stud. Myth. Slavica.

[19] Poursaleh Amiri S.M., Seyyed Ahmadi Zavieh S.S. (2022). Analytical Study of the Accompaniment of the Image of the Iris and Poppy Plant on the Zarrinfam Dishes of the Safavid Era from a Social Perspective. Negareh J..

[20] Lyte C. (1997). The Iris in History. A Guide to Species—Irises, Their Identification and Cultivation.

[21] Fărcaș C., Cristea V., Fărcaș S., Ursu T., Șuteu A., Roman A. (2015). The Symbolism of Garden and Orchard Plants and Their Representation in Paintings (I). Contrib. Bot..

[22] Dafni A., Lev E., Beckmann S., Eichberger C. (2006). Ritual Plants of Muslim Graveyards in Northern Israel. J. Ethnobiol. Ethnomedicine.

[23] Goody J. (1993). The Culture of Flowers.

[24] Lehner E., Lehner J. (2003). Folklore and Symbolism of Flowers, Plants, and Trees: With over 200 Rare and Unusual Floral Designs and Illustrations.

[25] Giner-Sorolla H. (2011). A Christian’s Treasury of Trees & Plants.

[26] Erken K., Gülbağ F., Erken S., Kaya E. (2013). The Adaptation of Turkish *Iris* L. Species to the Cultural Conditions. Acta Hortic..

[27] Goldblatt P., Manning J.C. (2008). The Iris Family: Natural History & Classification.

[28] Wilson C. (2006). Patterns in Evolution in Characters That Define Iris Subgenera and Sections. Aliso J. Syst. Florist. Bot..

[29] Brickell C. (2016). A-Z Encyclopedia of Garden Plants.

[30] Bresinsky A., Körner C., Kadereit J.W., Neuhaus G., Sonnewald U. (2013). Strasburger’s Plant Sciences.

[31] Meyer C.J., Peterson C.A., Steudle E. (2011). Permeability of *Iris germanica*’s Multiseriate Exodermis to Water, NaCl, and Ethanol. J. Exp. Bot..

[32] Stoie A., Vârban R. (2019). Botany: Morphology and Plant Anatomy Practical Works.

[33] Crișan I., Vidican R., Stoie A., Simea Ș.A. (2020). Spring-Autumn Arbuscular Mycorrhiza Colonization Dynamic in *Iris germanica* L. from Urban Microclimate. AgroLife Sci. J..

[34] Crișan I., Stoie A. (2021). Seasonal Arbuscular Mycorrhiza Colonization Dynamic Displays Genotype-Specific Pattern in *Iris sibirica* L.. Not. Sci. Biol..

[35] Crișan I., Vidican R., Stoian V., Vâtca S. (2019). Prospecting the Influence of Potting Substrate and Am Inoculation on *Iris pseudacorus* L.. Sci. Pap. Ser. Agron..

[36] Crișan I., Vidican R., Olar L., Stoian V., Morea A., Ștefan R. (2019). Screening for Changes on *Iris germanica* L. Rhizomes Following Inoculation with Arbuscular Mycorrhiza Using Fourier Transform Infrared Spectroscopy. Agronomy.

[37] Xing S., Zhang K., Hao Z., Zhang X., Chen B. (2023). Arbuscular Mycorrhizal Fungi Alter Arsenic Translocation Characteristics of *Iris tectorum* Maxim. J. Fungi.

[38] Zhu S., Mao H., Sun S., Yang X., Zhao W., Sheng L., Chen Z. (2025). Arbuscular Mycorrhizal Fungi Promote Functional Gene Regulation of Phosphorus Cycling in Rhizosphere Microorganisms of *Iris tectorum* under Cr Stress. J. Environ. Sci..

[39] Zhu S., Zhao W., Sun S., Yang X., Mao H., Sheng L., Chen Z. (2024). Metagenomic Analysis Revealed N-Metabolizing Microbial Response of *Iris tectorum* to Cr Stress after Colonization by Arbuscular Mycorrhizal Fungi. Ecotoxicol. Environ. Saf..

[40] Ranwala A.P., Miller W.B. (2008). Analysis of Nonstructural Carbohydrates in Storage Organs of 30 Ornamental Geophytes by High-Performance Anion-Exchange Chromatography with Pulsed Amperometric Detection. New Phytol..

[41] Crișan I., Cantor M. (2016). New Perspectives on Medicinal Properties and Uses of *Iris* sp.. Hop Med. Plants.

[42] Popovici P.C., Ancuceanu V.R., Dinu M. (2022). Mihaela Microscopic Characterization and Toxicological Assessment of *Iris germanica* L. Cultivated under Hydroponic and Geoponic Conditions. Farmacia.

[43] Shao L., Xu T., Wang X., Zhang R., Wang X., Ren Z., Zhang J., Xia Y., Li D. (2022). Integrative Comparative Assessment of Cold Acclimation in Evergreen and Deciduous *Iris* Species. Antioxidants.

[44] Royal Horticultural Society (RHS) Hardiness Ratings. http://www.rhs.org.uk/advice/rhs-hardiness-rating.

[45] Hočevar K., Vuleta A., Manitašević Jovanović S. (2025). Plastic Responses of *Iris pumila* Functional and Mechanistic Leaf Traits to Experimental Warming. Plants.

[46] Konarska A. (2022). Morphological, Anatomical, Ultrastructural, and Histochemical Study of Flowers and Nectaries of *Iris sibirica* L.. Micron.

[47] Crișan I., Vidican R., Oltean I., Stoie A., Stoian V. (2018). *Iris* Flower Visitors: Pollinators versus Nectar Thieves. Romanian J. Grassl. Forage Crops.

[48] Lozada-Gobilard S., Nielsen N., Sapir Y. (2023). Flower Size as an Honest Signal in Royal Irises (*Iris* Section *Oncocyclus*, Iridaceae). Plants.

[49] Zalmat A.S., Sotola V.A., Nice C.C., Martin N.H. (2021). Genetic Structure in Louisiana Iris Species Reveals Patterns of Recent and Historical Admixture. Am. J. Bot..

[50] Liu R., Gao Y., Guan C., Ding L., Fan Z., Zhang Q. (2023). The Comparison of Temporal Transcriptome Changes Between Morning-Opening and Afternoon-Opening Iris Flowers Reveals the Candidate Genes Regulating Flower Opening and Closing. J. Plant Biol..

[51] Wang Y., Zhang Y., Liu Q., Tong H., Zhang T., Gu C., Liu L., Huang S., Yuan H. (2021). Selection and Validation of Appropriate Reference Genes for RT-qPCR Analysis of Flowering Stages and Different Genotypes of *Iris germanica* L.. Sci. Rep..

[52] Fan Z., Gao Y., Guan C., Liu R., Wang S., Zhang Q. (2023). *FLOWERING LOCUS T* Homologue in Reblooming Bearded Iris (*Iris* Spp.) Plays a Role in Accelerating Flowering and Reblooming. S. Afr. J. Bot..

[53] Fan Z., Gao Y., Gao Y., Guan C., Liu R., Wang S., Zhang Q. (2023). Functional Characterization of Two Flowering Repressors *SHORT VEGETATIVE PHASE* and *TERMINAL FLOWER 1* in Reblooming Bearded Iris (*Iris* Spp.). Plant Sci..

[54] Roguz K., Gallagher M.K., Senden E., Bar-Lev Y., Lebel M., Heliczer R., Sapir Y. (2020). All the Colors of the Rainbow: Diversification of Flower Color and Intraspecific Color Variation in the Genus Iris. Front. Plant Sci..

[55] Bahreini Z., Abedi M., Ashori A., Parach A. (2024). Extraction and Characterization of Anthocyanin Pigments from Iris Flowers and Metal Complex Formation. Heliyon.

[56] Liu G., Liu H., Shi G., Xu N., Niu Z., Wang L., Zhao R., Wang L., Fan L. (2024). Multi-Omics Analysis of *Iris sanguinea* with Distinctive Flower Colors Provides Insights into Petal Coloration. Hortic. Plant J..

[57] Liu H., Shi G., Ye W., Behera J.R., Kilaru A., Wang L. (2025). Functional Role of *DFR* Genes in Various Blue *Iris* for the Regulation of Delphinidin Synthesis. Plant Physiol. Biochem..

[58] Zhou Z.-L., Wang G.-Y., Wang X.-L., Huang X.-J., Zhu Z.-S., Wang L.-L., Yang Y.-P., Duan Y.-W. (2023). Flower Color Polymorphism of a Wild Iris on the Qinghai-Tibet Plateau. BMC Plant Biol..

[59] Yuan Y., Sun Y., Zhao Y., Liu C., Chen X., Li F., Bao J. (2019). Identification of Floral Scent Profiles in Bearded Irises. Molecules.

[60] Cai K., Ban Z., Xu H., Chen W., Jia W., Zhu Y., Chen H. (2024). Analysis of Floral Scent Component of Three Iris Species at Different Stages. Horticulturae.

[61] Zhao Q., Li Y., Gu L., He D., Luo J., Zhang Y. (2024). Transcriptomic Profiling of the Floral Fragrance Biosynthesis of *Iris germanica* ‘Harvest of Memories’ and Functional Characterization of the *IgTPS14* Gene. Hortic. Plant J..

[62] Mitić B., Halbritter H., Šoštarić R., Nikolić T. (2013). Pollen Morphology of the Genus *Iris* L. (Iridaceae) from Croatia and Surrounding Area: Taxonomic and Phylogenetic Implications. Plant Syst. Evol..

[63] Choi T.-Y., Lee S.-R. (2024). Complete Plastid Genome of *Iris orchioides* and Comparative Analysis with 19 Iris Plastomes. PLoS ONE.

[64] Boltenkov E.V., Artyukova E.V. (2023). New Approach to the Systematics of the Section *Psammiris* (*Iris*, Iridaceae): What Does Chloroplast DNA Sequence Tell Us?. Plants.

[65] Crișan I., Vidican R., Stoian V., Stoie A. (2017). Wild *Iris* spp. from Romanian Meadows and Their Importance for Ornamental Plant Breeding. Rom. J. Grassl. Forage Crops.

[66] Volis S., Depalle F., Khassanov F., Yusupov Z., Deng T. (2024). *Oncocyclus* Irises: Phylogeny, Evolutionary History and Revised Taxonomy Based on Complete Chloroplast Genome Sequences. Plant Divers. Cent. Asia.

[67] Osmolovsky I., Shifrin M., Gamliel I., Belmaker J., Sapir Y. (2022). Eco-Geography and Phenology Are the Major Drivers of Reproductive Isolation in the Royal Irises, a Species Complex in the Course of Speciation. Plants.

[68] Saad L., Talhouk S.N., Mahy G. (2009). Decline of Endemic *Oncocyclus* Irises (Iridaceae) of Lebanon: Survey and Conservation Needs. Oryx.

[69] International Union for Conservation of Nature (IUCN) Red List of Threatened Species: *Iris*. https://www.iucnredlist.org/en.

[70] Boltenkov E.V., Artyukova E.V., Trias-Blasi A. (2021). Taxonomic Composition of *Iris* Subser. *Chrysographes* (Iridaceae) Inferred from Chloroplast DNA and Morphological Analyses. Plants.

[71] Boltenkov E., Artyukova E., Kozyrenko M., Erst A., Trias-Blasi A. (2020). *Iris sanguinea* Is Conspecific with *I. sibirica* (Iridaceae) According to Morphology and Plastid DNA Sequence Data. PeerJ.

[72] Wilson C.A., Boosalis Z., Sandor M., Crespo M.B., Martínez-Azorín M. (2023). Phylogeny of Species, Infraspecific Taxa, and Forms in *Iris* Subgenus *Xiphium* (Iridaceae), from the Mediterranean Basin Biodiversity Hotspot. Syst. Bot..

[73] Crișan I., Vidican R., Stoian V., Vâtcă S., Cantor M. Stomatal Index of Five *Iris × hollandica* Cultivars in Field Conditions. Proceedings of the International Conference on Life Sciences.

[74] Gaskin J.F., Pokorny M.L., Mangold J.M. (2016). An Unusual Case of Seed Dispersal in an Invasive Aquatic; Yellow Flag Iris (*Iris pseudacorus*). Biol. Invasions.

[75] Sennikov A., Khassanov F., Ortikov E., Kurbonaliyeva M., Tojibaev K.S. (2023). The Genus *Iris* L. s. l. (Iridaceae) in the Mountains of Central Asia Biodiversity Hotspot. Plant Divers. Cent. Asia.

[76] Cohen J.I., Turgman-Cohen S. (2023). The Conservation Genetics of *Iris lacustris* (Dwarf Lake Iris), a Great Lakes Endemic. Plants.

[77] Chirilă S.D., Vassilev K., Bădărău A.S. (2024). Wide Habitat Preference Found in a Rare, Regional Endemic Species: *Iris brandzae* Prodán (Iridaceae Juss., Subgenus *Limniris*, Series *Spuriae*) in Romania. Hacquetia.

[78] Mucioki M., Sowerwine J., Sarna-Wojcicki D., McCovey K., Bourque S.D. (2022). Understanding the Conservation Challenges and Needs of Culturally Significant Plant Species through Indigenous Knowledge and Species Distribution Models. J. Nat. Conserv..

[79] Othman Y.A., Ayasrah B., Al-Kofahi S. (2023). Habitat Selection to Reintroduce *Iris bismarckiana* in Semi-Arid Environments. Diversity.

[80] Volis S., Blecher M. (2022). Translocation Success in *Iris atrofusca*: Importance of Replicating Sites and Long-Term Monitoring. Restor. Ecol..

[81] Hurdu B.-I., Coste A., Halmagyi A., Szatmari P.-M., Farkas A., Pușcaș M., Dan Turtureanu P., Roșca-Casian O., Tănase C., Oprea A. (2022). Ex Situ Conservation of Plant Diversity in Romania: A Synthesis of Threatened and Endemic Taxa. J. Nat. Conserv..

[82] Botanic Gardens Conservation International (BGCI) PlantSearch: *Iris*. https://plantsearch.bgci.org/search?filter[genus]=Iris&sort=name.

[83] Gaoue O.G., Coe M.A., Bond M., Hart G., Seyler B.C., McMillen H. (2017). Theories and Major Hypotheses in Ethnobotany. Econ. Bot..

[84] Teixidor-Toneu I., Jordan F.M., Hawkins J.A. (2018). Comparative Phylogenetic Methods and the Cultural Evolution of Medicinal Plant Use. Nat. Plants.

[85] Purugganan M.D. (2019). Evolutionary Insights into the Nature of Plant Domestication. Curr. Biol..

[86] Wilson C.A., Padiernos J., Sapir Y. (2016). The Royal Irises (*Iris* Subg. *Iris* Sect. *Oncocyclus*): Plastid and Low-Copy Nuclea Data Contribute to an Understanding of Their Phylogenetic Relationships. Taxon.

[87] Belletti G., Fani E., Marescotti A., Scaramuzzi S. (2013). The Role of Traditional Products in the Valorisation of Marginal Rural Areas: The Case of *Iris pallida*. Span. J. Rural Dev..

[88] Samad N.A., Hidalgo O., Saliba E., Siljak-Yakovlev S., Strange K., Leitch I.J., Dagher-Kharrat M.B. (2020). Genome Size Evolution and Dynamics in *Iris*, with Special Focus on the Section *Oncocyclus*. Plants.

[89] Mykhailenko O., Buydin Y., Ivanauskas L., Krechun A., Georgiyants V. (2022). Innovative GACP Approaches for Obtaining the Quality *Iris hybrida* Leaves for the Pharmaceutical Industry. Chem. Biodivers..

[90] Bruccoleri R.E., Oakeley E.J., Faust A.M.E., Altorfer M., Dessus-Babus S., Burckhardt D., Oertli M., Naumann U., Petersen F., Wong J. (2023). Genome Assembly of the Bearded Iris, *Iris pallida* Lam. GigaByte.

[91] Ghasemi G., Ayyari M., Azimi M.-H., Ebadi M.-T. (2023). Orris Root Diversity and Quality Assessment: Multivariate Analysis of Phytochemicals and Antioxidant Properties. Ind. Crops Prod..

[92] National Center for Biotechnology Information *Iris* Genome. https://www.ncbi.nlm.nih.gov/datasets/genome/?taxon=26378.

[93] The International Union for the Protection of New Varieties of Plants (UPOV) Guidelines for the Conduct of Tests for Distinctness, Uniformity and Stability for Iris (Bulbous) (Iris L.) 2000. https://www.upov.int/edocs/tgdocs/en/tg174.pdf.

[94] Noli J., Nichols B., Rivarola A., White G., Snyder G., Rose L., Strauss D., Rieniets K., Markham S., Vaughn K. (2024). Handbook for Judges and Show Officials.

[95] Ding L., Liu R., Gao Y., Xiao J., Lv Y., Zhou J., Zhang Q. (2023). Effect of Tetraploidization on Morphological and Fertility Characteristics in *Iris* × *norrisii* Lenz. Sci. Hortic..

[96] Zhao X., Zhang X., Wu Y., Yu F., Su B., Li X., Huang D. (2024). Cross Compatibility and Endogenous Phytohormone Profiles in Interspecific Hybridization between *Iris tectorum* and *Iris germanica*. Sci. Hortic..

[97] Crişan I., Stoie A., Cantor M. (2016). Overwintering of Some Hardy Iris Species in Agro-Botanical Garden UASVM Cluj-Napoca. Agricultura.

[98] Muntean L.S., Tămaș M., Muntean S., Muntean L., Duda M.M., Vârban D.I., Florian S. (2016). Treatise of Cultivated and Spontaneous Medicinal Plants.

[99] Arditti J., Pray T.R. (1969). Dormancy Factors in *Iris* (Iridaceae) Seeds. Am. J. Bot..

[100] Abubakar M.S., Attanda M.L., Abubakar M.S., Attanda M.L. (2022). Factors That Cause Seed Dormancy. Seed Biology Updates.

[101] Śmigała-Lasota M., Dziurka K., Dąbrowska A., Winiarczyk K. (2023). Development of the Male and Female Gametophyte, Fertilization, and Assessment of Germination and Regulation of Dormancy in *Iris aphylla* L. Seeds. Acta Sci. Pol. Hortorum Cultus.

[102] Yoon M.J., Kim S.H., Gil M., Park E.H., Oh S.I., Lee S.Y., Ko C.H. (2022). Seed Dormancy and Germination in *Iris laevigata* (Iridaceae), A Rare Species in Korea. Flower Res. J..

[103] Pianova A.S., Berdasova K.S., Mironova L.N., Salokhin A.V., Sabutski Y.E. (2025). Optimization of Seed Scarification Protocol and Assessment of the Effect of 6-BAP Concentration on Microplants of *Iris mandshurica* Maxim. (Iridaceae). Bot. Pacifica.

[104] Gillard M.B., Castillo J.M., Mesgaran M.B., Futrell C.J., Grewell B.J. (2022). Germination Niche Breadth of Invasive *Iris pseudacorus* (L.) Suggests Continued Recruitment from Seeds with Global Warming. Am. J. Bot..

[105] Volis S. (2024). Effect of Intra-Seasonal Variation in Precipitation on Seed Germination and Seedling Growth in *Iris atrofusca*: Implications for Conservation. Isr. J. Ecol. Evol..

[106] Park H.B., Lee B.-D., Lee C.W., Hwang J.E., Park H.J., Kim S., An J., Kim P.B., Kim N.Y. (2021). Germination Characteristics and Seed Dormancy of *Iris dichotoma* Pall., an Endangered Species Native to Korea. Proc. Natl. Inst. Ecol. Repub. Korea.

[107] Hirai H., Mori G. (1996). Forcing Culture of Freesia and Dutch Iris Using Spot Cooling System.

[108] Hellivan P.-J. (2009). Orris: A Star of Inspiration. Perfum. Flavorist.

[109] Meucci A., Ghelardi C., Maggini R., Malorgio F., Chietera G., Mensuali A. (2024). Micropropagation via Somatic Embryogenesis of *Iris pallida* Lam. Ecotypes. Plant Cell Tissue Organ Cult. PCTOC.

[110] Meucci A., Ghelardi C., Chietera G., Mensuali A. (2024). Synthetic Seed Production and Slow Growth Storage of In Vitro Cultured Plants of *Iris pallida* Lam. Horticulturae.

[111] Verma V., Kumar A., Thakur M., Bhargava B., Priti S. (2022). Meta-Topolin Mediated in Vitro Propagation in an Ornamentally Important Crop *Iris × hollandica* Tub. Cv. Professor Blaauw and Genetic Fidelity Studies Using SCoT Markers. Plant Cell Tissue Organ Cult. PCTOC.

[112] Yasemin S., Beruto M. (2024). A Review on Flower Bulb Micropropagation: Challenges and Opportunities. Horticulturae.

[113] Jevremović S., Milutinović M., Veličković K., Gašić U., Škoro N., Puač N., Živković S. (2025). Cold Plasma Treatment Alters the Morphology, Oxidative Stress Response and Specialized Metabolite Content in Yellow Iris (*I. reichenbachii*) Callus. Horticulturae.

[114] Niketić M., Tomović G., Siljak-Yakovlev S. (2018). A New Spontaneous Hybrid between the Cultivated and Wild Iris Species from Serbia. Bull. Nat. Hist. Mus..

[115] Beresford-Kroeger D. (2004). A Garden for Life: The Natural Approach to Designing, Planting, and Maintaining a North Temperate Garden.

[116] Fan Z., Gao Y., Guo L., Cao Y., Liu R., Zhang Q. (2019). Phenotypic Variations and Heritability in Hybrid Populations of Bearded Iris. HortScience.

[117] Asgari E., Taghizadeh M., Abbasifar A. (2022). Exploration and Morphologic Variation of Iris Wild Species with Ornamental Potential. Ornam. Hortic..

[118] Kemper William Centre for Home Gardening Iris Fact Sheet, Missouri Botanical Garden. https://www.missouribotanicalgarden.org/Portals/0/Gardening/Gardening%20Help/Factsheets/Iris22.pdf.

[119] Crișan I., Vidican R., Plesa A., Mihaiescu T. (2021). Phytoremediation Potential of *Iris* spp.. Bull. Univ. Agric. Sci. Vet. Med. Cluj-Napoca Agric..

[120] Sandenbergh E., Gervazoni P., Grewell B., Franceschini C., Minuti G., McGrannachan C., Stiers I., Coetzee J. (2025). Biology of Invasive Plants 7. *Iris pseudacorus* L. (Iridaceae). Invasive Plant Sci. Manag..

[121] Bilgaiyan P., Shivhare N., Gowripathi Rao N.R.N.V. (2023). Phytoremediation of Wastewater through Implemented Wetland—A Review. Proceedings of the 2023 International Conference on Sustainable Technologies in Civil and Environmental Engineering (ICSTCE 2023).

[122] Sarti C., Cincinelli A., Bresciani R., Rizzo A., Chelazzi D., Masi F. (2024). Microplastic Removal and Risk Assessment Framework in a Constructed Wetland for the Treatment of Combined Sewer Overflows. Sci. Total Environ..

[123] Yadav N., Govindwar S.P., Rane N., Ahn H.-J., Xiong J.-Q., Jang M., Kim S.H., Jeon B.-H. (2021). Insights on the Role of Periphytic Biofilm in Synergism with *Iris pseudacorus* for Removing Mixture of Pharmaceutical Contaminants from Wastewater. J. Hazard. Mater..

[124] Chen X., Zhong F., Chen Y., Wu J., Cheng S. (2022). The Interaction Effects of Aeration and Plant on the Purification Performance of Horizontal Subsurface Flow Constructed Wetland. Int. J. Environ. Res. Public Health.

[125] Larson R.A. (2013). Introduction to Floriculture.

[126] Dole J.M. (2003). Research Approaches for Determining Cold Requirements for Forcing and Flowering of Geophytes. HortScience.

[127] Mortazavi H., Hassanpour Asil M. (2010). Effects of Temperature and Gibberellic Acid on Forcing and Quality Improvement of Iris (*Iris hollandica* Cv. ‘Blue Magic’) Cut Flowers. J. Agric. Sci. Sustain. Prod..

[128] Elphinstone E.D., Rees A.R., Atherton J.G. (1990). Temperature and Development in *Iris x hollandica* during Pre-Planting Storage. II. Floral Initiation. J. Hortic. Sci..

[129] Janick J. (2010). Horticultural Reviews, Volume 36.

[130] Seraya L.G., Larina G.E., Bondareva E.V., Ivanova I.O., Polyakova N.N. (2021). Ecological Methods of Protecting Bearded Irises in the Urban Environment. IOP Conf. Ser. Earth Environ. Sci..

[131] Crișan I. (2020). Study on Dynamic and Effects of Arbuscular Mycorrhizae in Some Ornamentals from Cluj Conditions. Ph.D. Thesis.

[132] Yadav P., Yadav K., Mishra A., Singh K. (2024). An Assessment and Analysis of Diseases of Economically Important Plant Members of Family Iridaceae. J. Plant Dis. Prot..

[133] Dugan F.M., Lupien S.L., Vahling-Armstrong C.M., Chastagner G.A., Schroeder B.K. (2014). Host Ranges of North American Isolates of *Penicillium* Causing Blue Mold of Bulb Crops. Crop Prot..

[134] Wang C., Jiang N., Zhu Y., Xue H., Piao C., Li Y. (2022). *Colletotrichum truncatum* Causing Anthracnose Disease of *Iris lactea* in Beijing, China. J. Phytopathol..

[135] Lu X., Wang Y., Dong A., Liu X., Diao G., Jia N., Zhu T. (2024). First Report of *Alternaria alternata* Causing Fruit Blight Disease on *Iris sanguinea* in China. Plant Dis..

[136] Oxspring S., Carroll S., Bryning A., Gaunt A., Aspin A. (2024). First Report of *Pseudomonas syringae* Infecting *Iris foetidissima* in the United Kingdom. New Dis. Rep..

[137] Karanfil A., Korkmaz S. (2022). First Report of Iris Severe Mosaic Virus in Bulbous Irises in Turkey. J. Plant Pathol..

[138] Grunwald D.J., Stroschein S.M., Grinstead S., Mollov D., Rioux R.A., Rakotondrafara A.M. (2023). Targeting the Highly Conserved 3′ Untranslated Region of Iris Severe Mosaic Virus for Sensitive Monitoring of the Disease Prevalence in Iris Production. Plant Dis..

[139] Bag S., Schwartz H.F., Cramer C.S., Havey M.J., Pappu H.R. (2015). Iris Yellow Spot Virus (Tospovirus: Bunyaviridae): From Obscurity to Research Priority. Mol. Plant Pathol..

[140] Doyon J., Savage J., Bailleul S., Labelle S., Brodeur J. (2022). Susceptibility of Iris (Iridaceae) to Larval Infestation by *Neorthacheta dissimilis* (Diptera: Scathophagidae). Can. Entomol..

[141] Vuleta A., Hočevar K., Manitašević Jovanović S., Raičević J., Plećaš M. Assessment of *Iris pumila* L. Pollinator and Florivore Diversity in a Common Garden: A Pan-Trap Experiment. Proceedings of the 4th International Conference on Plant Biology [and] 23rd SPPS Meeting.

[142] Minuti G., Coetzee J.A., Stiers I. (2023). Contrasting Effects of Climate Change on the Invasion Risk and Biocontrol Potential of the Invasive *Iris pseudacorus* L. between Northern and Southern Hemisphere. Biol. Control.

[143] Minuti G., Coetzee J.A., Ngxande-Koza S., Hill M.P., Stiers I. (2021). Prospects for the Biological Control of *Iris pseudacorus* L. (Iridaceae). Biocontrol Sci. Technol..

[144] Iwashina T., Mizuno T. (2020). Flavonoids and Xanthones From the Genus Iris: Phytochemistry, Relationships with Flower Colors and Taxonomy, and Activities and Function. Nat. Prod. Commun..

[145] Yan H., Lyu H., Otgon O., Lu J., Peng D., Zhu Y., Jiang J. (2024). Five New Flavonoids and Their Pharmacological Activities from *Iris tenuifolia* Pall. Fitoterapia.

[146] Jaegerova T., Zlechovcova M., Benes F., Kronusova O., Kastanek P., Hajslova J. (2024). Investigation of *Iris versicolor* Metabolic Profile and Optimization of the Isolation of Bioactive Components on a Semi-Operation Scale. Process Biochem..

[147] Galfré A., Martin P., Petrzilka M. (1993). Direct Enantioselective Separation and Olfactory Evaluation of All Irone Isomers. J. Essent. Oil Res..

[148] Brenna E., Fuganti C., Serra S. (2008). Applications of Biocatalysis in Fragrance Chemistry: The Enantiomers of α-, β-, and γ-Irones. Chem. Soc. Rev..

[149] Yamazaki S., Miyazaki M. (2025). Synthesis and Olfactory Evaluation of (±)-β-Irone. Sci. Rep..

[150] Inoue T., Kiyota H., Oritani T. (2000). Synthesis of Both Enantiomers of *cis*-α-Irone and *cis*-γ-Irone, Principal Constituents of Iris Oil, via Resolution of (±)-2,2,4-Trimethyl-3-Cyclohexene-1-Carboxylic Acid. Tetrahedron Asymmetry.

[151] Chen X., T R., Esque J., Zhang C., Shukal S., Lim C.C., Ong L., Smith D., André I. (2022). Total Enzymatic Synthesis of cis-α-Irone from a Simple Carbon Source. Nat. Commun..

[152] Brenna E., Fuganti C., Ronzani S., Serra S. (2001). Enzyme-Mediated Syntheses of the Enantiomers of γ-Irones. Helv. Chim. Acta.

[153] Živković U., Avramov S., Miljković D., Barišić Klisarić N., Tubić L., Mišić D., Šiler B., Tarasjev A. (2021). Genetic and Environmental Factors Jointly Impact Leaf Phenolic Profiles of *Iris variegata* L.. Plants.

[154] Mykhailenko O., Chetvernya S., Bezruk I., Buydin Y., Dhurenko N., Palamarchuk O., Ivanauskas L., Georgiyants V. (2022). Bioactive Constituents of *Iris hybrida* (Iridaceae): Processing Effect. Biomed. Chromatogr..

[155] Palchetti E., Brilli L., Padovan G., Mariani G., Marini L., Moretta M. (2025). Effect of Planting Density and Harvesting Age on *Iris pallida* Lam. Biomass, Morphology and Orris Concrete Production. Agronomy.

[156] Ieri F., Vignolini P., Urciuoli S., Pinelli P., Romani A. (2024). The Cultivation of *Iris pallida* as an Opportunity for the Enhancement of Tuscan Agro-Biodiversity and a Resource for the Local Economy. Innovation, Quality and Sustainability for a Resilient Circular Economy.

[157] Pezzarossa B., Borghesi E., Pini R., Bretzel F., Maggini R., Malorgio F. (2020). Department of Agriculture, Food and Environment, University of Pisa, Pisa, Italy Influence of Pedo-Climatic Conditions on the Quality of *Iris pallida* Rhizomes. Eur. J. Hortic. Sci..

[158] Ghasemi G., Ayyari M., Azimi M.-H., Ebadi M.-T. (2025). Rapid Alternative of the Post-Harvest Process to Enhance the Irones Content in *Iris germanica* L.. J. Agric. Food Res..

[159] Kara N., Baydar H. (2014). Scent Components in Essential Oil, Resinoids and Absolute of Iris (*Iris florentina* L.). Anadolu J. Agric. Sci..

[160] Kara N., Gürbüzer G. (2019). Effect of Harvest Times on Rhizoma Yield, Essential Oil Content and Composition in *Iris germanica* L. Species. Turk. J. Agric.—Food Sci. Technol..

[161] Roger B., Jeannot V., Fernandez X., Cerantola S., Chahboun J. (2012). Characterisation and Quantification of Flavonoids in *Iris germanica* L. and *Iris pallida* Lam. Resinoids from Morocco. Phytochem. Anal..

[162] Orris Oil 8002-73-1. https://www.chemicalbook.com/ChemicalProductProperty_EN_CB3187265.htm.

[163] Karpitskiy D.A., Bessonova E.A., Shishov A.Y., Kartsova L.A. (2024). Selective Extraction of Plant Bioactive Compounds with Deep Eutectic Solvents: *Iris sibirica* L. as Example. Phytochem. Anal..

[164] The Committee on Herbal Medicinal Products (HMPC), Good Agricultural and Collection Practice (GACP) for Starting Materials of Herbal Origin, European Medicines Agency. https://www.ema.europa.eu/en/documents/scientific-guideline/guideline-good-agricultural-collection-practice-gacp-starting-materials-herbal-origin-revision-1_en.pdf.

[165] Cherver T., Gonçalves A., Lepeule C. (2022). Farm Certification Schemes for Sustainable Agriculture—State of Play and Overview in the EU and in Key Global Producing Countries, Concepts and Methods.

[166] Pan J., He S., Zheng J., Shao J., Li N., Gong Y., Gong X. (2019). The Development of an Herbal Material Quality Control Strategy Considering the Effects of Manufacturing Processes. Chin. Med..

[167] Mykhailenko O., Ivanauskas L., Bezruk I., Petrikaitė V., Georgiyants V. (2022). Application of Quality by Design Approach to the Pharmaceutical Development of Anticancer Crude Extracts of *Crocus sativus* Perianth. Sci. Pharm..

[168] (2024). Oils of Orris Rhizome (*Iris pallida* Lam. or *Iris germanica* L.)—Determination of Irone Content—Method Using Gas Chromatography on a Capillary Column.

[169] European Directorate for the Quality of Medicines & HealthCare of the Council of Europe (2009). European Pharmacopoeia.

[170] Gooderham N.J., Cohen S.M., Eisenbrand G., Fukushima S., Guengerich F.P., Hecht S.S., Rietjens I.M.C.M., Rosol T.J., Davidsen J.M., Harman C.L. (2023). FEMA GRAS Assessment of Natural Flavor Complexes: Sage Oil, Orris Root Extract and Tagetes Oil and Related Flavoring Ingredients. Food Chem. Toxicol..

[171] Rogers A. (2021). Going Back to the Roots: A Phytochemical Investigation of the Use of Iris in the Ancient Mediterranean. Ph.D. Thesis.

[172] Pliny T.E. Pliny Natural History: Book XXI: Chapter IXX 100 AD. https://www.gutenberg.org/files/61113/61113-h/61113-h.htm#BOOK_XXI_CHAP_19.

[173] Eastaugh N., Walsh V., Chaplin T., Siddall R. (2004). The Pigment Compedium: A Dictionary of Historical Pigments.

[174] Oztas F., Turkmen A., Oztas H., Turkmen M. (2024). The Medical Properties of Iris and Its Usage in Pharmaceutical, Perfumery and Cosmetic Industries. Med. Res. Its Appl..

[175] Lim T.K., Lim T.K. (2016). *Iris x germanica*. Edible Medicinal and Non-Medicinal Plants: Volume 11 Modified Stems, Roots, Bulbs.

[176] Yeon J., Suh S., Youn U., Bazarragchaa B., Enebish G., Seo J. (2021). Methanol Extract of Mongolian *Iris bungei* Maxim. Stimulates 3T3-L1 Adipocyte Differentiation. J. Nanosci. Nanotechnol..

[177] Briot E. (2011). From Industry to Luxury: French Perfume in the Nineteenth Century. Bus. Hist. Rev..

[178] Hoang L., Beneš F., Fenclová M., Kronusová O., Švarcová V., Řehořová K., Baldassarre Švecová E., Vosátka M., Hajšlová J., Kaštánek P. (2020). Phytochemical Composition and In Vitro Biological Activity of *Iris* spp. (Iridaceae): A New Source of Bioactive Constituents for the Inhibition of Oral Bacterial Biofilms. Antibiotics.

[179] Medic B.S., Mujkić A.J.-, Cubara B., Pasic A.D.-, Kurtovic J.H., Bajrović K., Omeragić E., Dedić M., Bogunić F., Pojskic L. (2024). Crude Extracts of Three *Iris* Species as Sources of MRSA Antimicrobial Compounds. Eur. J. Biol..

[180] Abdel-Baki P.M., El-Sherei M.M., Khaleel A.E., Abdel-Aziz M.M., Okba M.M. (2022). Irigenin, a Novel Lead from *Iris confusa* for Management of *Helicobacter pylori* Infection with Selective COX-2 and HpIMPDH Inhibitory Potential. Sci. Rep..

[181] Unver T., Uslu H., Gurhan I., Goktas B. (2024). Screening of Phenolic Components and Antimicrobial Properties of *Iris persica* L. Subsp. Persica Extracts by in Vitro and in Silico Methods. Food Sci. Nutr..

[182] Lê H.G., Hwang B.S., Choi J.-S., Jeong Y.T., Kang J.-M., Võ T.C., Oh Y.T., Na B.-K. (2024). Iris Setosa Pall. Ex Link Extract Reveals Amoebicidal Activity against *Acanthamoeba castellanii* and *Acanthamoeba polyphaga* with Low Toxicity to Human Corneal Cells. Microorganisms.

[183] Kumar R., Bhattacharjee A., Tiwari S. (2022). Plant-Derived Ribosome-Inactivating Proteins Involved in Defense against Plant Viruses. Eur. J. Plant Pathol..

[184] Potdar M.B., Patil K., Usman M.R.M., Wadekar R.R. (2025). An Update on the Role of Antioxidants in Health and Disease Prevention. Antioxidants as Nutraceuticals.

[185] Budzianowska A., Banaś K., Budzianowski J., Kikowska M. (2025). Antioxidants to Defend Healthy and Youthful Skin—Current Trends and Future Directions in Cosmetology. Appl. Sci..

[186] Gulcin İ. (2025). Antioxidants: A Comprehensive Review. Arch. Toxicol..

[187] Amin H.I.M., Hussain F.H.S., Najmaldin S.K., Thu Z.M., Ibrahim M.F., Gilardoni G., Vidari G. (2021). Phytochemistry and Biological Activities of Iris Species Growing in Iraqi Kurdistan and Phenolic Constituents of the Traditional Plant *Iris postii*. Molecules.

[188] Yehia S.M., Ayoub I.M., Watanabe M., Devkota H.P., Singab A.N.B. (2023). Metabolic Profiling, Antioxidant, and Enzyme Inhibition Potential of *Iris pseudacorus* L. from Egypt and Japan: A Comparative Study. Sci. Rep..

[189] Chandni, Ahmad S.S., Saloni A., Bhagat G., Ahmad S., Kaur S., Khan Z.S., Kaur G., Abdi G. (2024). Phytochemical Characterization and Biomedical Potential of *Iris kashmiriana* Flower Extracts: A Promising Source of Natural Antioxidants and Cytotoxic Agents. Sci. Rep..

[190] Kim J.-S., Lee H.-J., Yoon E.-J., Lee H., Ji Y., Kim Y., Park S.-J., Kim J., Bae S. (2023). Protective Effect of *Iris germanica* L. Rhizome-Derived Exosome against Oxidative-Stress-Induced Cellular Senescence in Human Epidermal Keratinocytes. Appl. Sci..

[191] Bilal M., Naz A., Khan A., Salman, Ghaffar R., Abrar A. (2023). Assessment of *Iris albicans* Lange as Potential Antimicrobial and Analgesic Agent. PLoS ONE.

[192] Michalak A., Krauze-Baranowska M., Migas P., Kawiak A., Kokotkiewicz A., Królicka A. (2021). *Iris pseudacorus* as an Easily Accessible Source of Antibacterial and Cytotoxic Compounds. J. Pharm. Biomed. Anal..

[193] Tie F.-F., Fu Y.-Y., Hu N., Chen Z., Wang H.-L. (2022). Isolation of Oligostilbenes from *Iris lactea* Pall. var. *chinensis* (Fisch.) Koidz and Their Anti-Inflammatory Activities. RSC Adv..

[194] Abdullah F.O. (2024). Phytochemical Identification by LC-ESI MS/MS Method of the *Iris barnumiae* Methanolic Extract and Its Antiproliferative and Apoptosis-Inducing Effects. Biomass Convers. Biorefinery.

[195] Ranđelović D., Jakovljević K., Zeremski T., Pošćić F., Baltrėnaitė-Gedienė E., Noulas C., Mašková P., Jurković J., Baragaño Coto D., Milićević T. (2025). Phytoremediation Potential of Metallophytes in Europe: Progress, Enhancement Strategies, and Biomass Utilisation. J. Environ. Manag..

[196] Greksa A., Mihajlović I., Ljubojević M., Blagojević B., Vijuk M.I., Podunavac-Kuzmanović S., Kovačević S., Štrbac M.P. (2024). Investigation of *Juncus* and *Iris* Plant Potential—Two Native Serbian Species for Utilization in Nature-Based Solutions towards Improving the Quality of Water Contaminated with Zinc and Supporting Biodiversity. Sustainability.

[197] Zhao W., Chen Z., Yang X., Sheng L., Mao H., Zhu S. (2023). Metagenomics Reveal Arbuscular Mycorrhizal Fungi Altering Functional Gene Expression of Rhizosphere Microbial Community to Enhance *Iris tectorum*’s Resistance to Cr Stress. Sci. Total Environ..

[198] Nguyen T.T., Huang H., Oda M., Soda S. (2024). Constructed Wetlands Planted with Iris for Treatment of Wastewater Simulating a Typical Mine Drainage in Japan: Effects of Organic-Feeding on Removal of Zn and Cd. J. Asia-Jpn. Res. Inst. Ritsumeikan Univ..

[199] Brunhoferova H., Venditti S., Laczny C.C., Lebrun L., Hansen J. (2022). Bioremediation of 27 Micropollutants by Symbiotic Microorganisms of Wetland Macrophytes. Sustainability.

[200] Li T., Wang Y., Niu Y., Zhang Z., Liu J., Wang X., Wang J., Li J., Wang L. (2025). Enhanced Cadmium Adsorption Mechanisms Utilizing Biochar Derived from Different Parts of Wetland Emergent Plants *Iris sibirica* L.. Processes.

[201] Yu J., Lee J.-H., Song M.-H., Keum Y.-S. (2023). Metabolomic Responses of Lettuce (*Lactuca sativa*) to Allelopathic Benzoquinones from Iris Sanguinea Seeds. J. Agric. Food Chem..

[202] Sothearith Y., Appiah K.S., Sophea C., Smith J., Samal S., Motobayashi T., Fujii Y. (2024). Influence of β-Ionone in the Phytotoxicity of the Rhizome of *Iris pallida* Lam. Plants.

[203] Shurigin V., Alimov J., Davranov K., Gulyamova T., Egamberdieva D. (2022). The Diversity of Bacterial Endophytes from *Iris pseudacorus* L. and Their Plant Beneficial Traits. Curr. Res. Microb. Sci..

[204] Oloumi H., Khaleghi M., Dalvand A. (2023). Isolation and Identification of Endophytic Actinobacteria from *Iris persica* and *Echium amoenum* Plants and Investigation of Their Effects on Germination and Growth of Wheat Plant. Food Sci. Nutr..

